# Deciphering the influence of fertilization systems on the *Allium ampeloprasum* rhizosphere microbial diversity and community structure through a shotgun metagenomics profiling approach

**DOI:** 10.1186/s40793-025-00771-w

**Published:** 2025-10-06

**Authors:** Oluwaseun Emmanuel Shittu, Ben Jesuorsemwen Enagbonma, Olubukola Oluranti Babalola

**Affiliations:** 1 Food Security and Safety Focus Area, Faculty of Natural and Agricultural Sciences, North-West University, Private Mail Bag X2046, Mmabatho, 2735 South Africa; 2 Department of Life Sciences, Imperial College London, Silwood Park Campus, Buckhurst Road, Ascot, Berkshire, SL5 7PY UK

**Keywords:** Fertilizers, Leek, Food security, Rhizosphere microbiome, Shotgun sequencing, Synthetic fertilizer

## Abstract

**Background:**

Chemical fertilizer application in agriculture over the years has been a vital instrument to boost agricultural yields and soil fertility, but has threatened the diversity of the rhizosphere microbiomes in the soil. However, knowledge about the impacts of biofertilizers (BF) as well as chemical fertilizers (CF) on *Allium ampeloprasum* rhizosphere’s microbiomes is still limited. Hence, this study investigated the metagenomic profiling of *A. ampeloprasum* rhizosphere under different fertilization systems and in bulk soils, to obtain a depiction of their associated microbial diversity and community structure, which will inform best agricultural practices.

**Method:**

The entire DNA sample was mined from soil samples taken from an independent uncultivated bulk soil and the rhizosphere of *A. ampeloprasum* treated with chemical and biofertilizer and subjected to shotgun metagenomics sequencing.

**Results:**

The taxonomic analysis of our metagenome unveiled that while all soil samples exhibited similar core microbial phyla, *Bacteroidota* and *Verrucomicrobiota* were exclusive to the biofertilizer (G2) plot. *Actinobacteria* and *Pseudomonadota* (*Proteobacteria*) were predominant in the biofertilizer plot (G2), chemical fertilizer (G1), and bulk soil (G3) plots, respectively. Genera such as *Dyadobacter, Verrucomicrobium, Streptomyces,* and *Haliangium* were exclusively detected in the biofertilizer plot (G2). Alpha diversity analysis showed that G2 harboured the most diverse microbial community, followed by G3, with the lowest diversity found in the G1 plot, highlighting the importance of biofertilizer in increasing microbial diversity.

The observed differences in the microbial diversity and community structure are highly linked to the nature of fertilizer applied and the distinct physicochemical parameters of the three plots. However, redundancy analysis subsequently highlighted total nitrogen and carbon as the key environmental influencers impacting the microbial community structure and composition.

**Conclusion:**

This study underscores the potential of biofertilizers in boosting the rhizosphere microbial diversity, improving soil health, and offer a sustainable alternative to chemical fertilizers, thereby supporting long-term agricultural sustainability and resilience in food production systems.

**Supplementary Information:**

The online version contains supplementary material available at 10.1186/s40793-025-00771-w.

## Introduction

The purpose of the Goal 2 of United Nations (UN) 2030 Agenda for Sustainable Development Goals (SDGs) is to end hunger, advance sustainable agriculture, improve nutrition, and ensure food security [54]. To achieve this goal, farmers resort to the application of synthetic inputs, which improve growth yield, but with regards to public health, the environment and nutrition, this poses a great risk [62]. In terms of environmental risk, this has affected the balance of biodiversity in host plant environments [89]. These drawbacks led to the instigation of alternative measures of achieving food safety and security in a feasible and continuous manner, involving biofertilizer application, which leads to the stabilization of the microbial growth rate. To understand the impact of these fertilizer inputs on biodiversity in the rhizosphere, the rhizosphere of various crops has been the subject of numerous investigations. Du et al. [37] stated that synthetic or chemical fertilizer and biofertilizer inputs affect microbiome diversity by inducing bacterial and archaeal predominance, respectively. In the case of Allium families, these shifts in microbiome composition could have a significant effect, as Allium plants are recognized to be a prodigious home of various vitamins, including vitamins A, C, and K, as well as a number of B vitamins, including B6 and folate. Additionally, these plants also contain antioxidant properties and are high in dietary fiber when consumed as vegetables, making them important in food nutrition [116]. Beyond their vitamin content, their nutritional profile and active compounds like Allicine, flavonoids, and sulphur compounds coupled with their traditional importance in appetite suppression and improvement of digestive health, make it highly valuable [93]. However, the cumulative influence of their potential impacts on diseases (like cardiovascular, cancer, and neurodegenerative diseases), and malnutrition prevention, coupled with their antimicrobial properties, which help combat microbial infections, and their impacts on immune system support, can add to overall health [11]. On a larger scale, such improvements can positively impact public health by reducing disease prevalence, improving quality of life, and alleviating healthcare burdens. Hence, it is critical to comprehend the degree of biodiversity changes as a result of fertilization practices aimed at enhancing *A. ampeloprasum* production and increasing yield.

The microbial population of the rhizosphere is composed of viruses, fungi, bacteria, and, archaea with bacteria being the most prevalent [91]. Understanding how agricultural practices farmers adopt to improve growth and, influence their proliferation or reduction is a positive step toward attaining agro-ecosystem sustainability. Presently, there is a global degradation and loss of soil biodiversity owing to the indiscriminate introduction of chemical fertilizers. The introduction of rhizosphere beneficial microbiomes could make Goal 2 of the SDGs achievable by the year 2030 by alleviating hunger and making food available for all [38].

In this context, embracing an eco-friendly, sustainable, cost-effective, and alternative source of fertilizers called biofertilizer could eradicate all the major problems posed by chemical fertilizers and boost agricultural production [71]. Biofertilizer function as a major contributor to sustainable farming by helping plants solubilize insoluble nutrients to improve soil fertility, offering protection against diseases and drought, and increasing plant tolerance and hence crop production [7].

In addition, biofertilizers contribute to mitigating the occurrence of soil-borne and crop diseases as they considerably improve the community of beneficial microbes in the soil ecology. These beneficial microorganisms including bacteria, archaea, and fungi effectively suppress pathogenic organisms by influencing them in many ways like competition of nutrients and niche space, producing antimicrobial compounds and by induction of systemic resistance in the plants [21]. Moreover, biofertilizers make the soil unfriendly to the pathogens even when they are in the soil thus resulting in healthier plants with increased resistance to diseases due to the presence of the beneficial microbial community [28, 33].

Over time, fertilization systems have been proven to significantly impact the soil functional genes by altering the composition of the microbial community and their associated functions involved in the cycling of nutrients which in turn affects the soil health and crop productivity. The traditional chemical fertilizers have been demonstrated to interfere with the microbial diversity and associated nutrient turnover process of genes [41]. Conversely, biofertilizers have been found to be a potential sustainable solution that can boost the diversity of beneficial microbes, functional expression of genes to facilitate nutrient release, and soil fertility [45, 46, 100].

Profiling the microbial community organization and diversity related to *A. ampeloprasum*’*s* rhizosphere under different fertilization systems using a shotgun sequencing approach is an essential milestone in comprehending the complex impacts of agro-chemical fertilizers as well as biofertilizer on microbial ecosystems which carry out an essential task regarding the health of plants, sustainable agriculture and fertility of soil.

The rhizosphere itself is a home to a vast array of microorganisms, both culturable as well as unculturable [88]. Many of these core microbiomes are recognized for their tasks regarding the growth and health of plants. Understanding these core microbiomes is important in developing methods to improve the crop’s resilience and yield [1].

Additionally, the applications of biofertilizers will ensure the long-term fertility, crop productivity of the soil and also contributes to a significant decrease in the use of chemical or synthetic fertilizers [58].

Among the various fertilization methods utilized in agriculture, the treatment of plants with organic fertilizers (biofertilizers) are recognized for enhancing and boosting the diversity of microorganisms and the community structure within agricultural lands. Supporting this, a metagenomics study on the impact of inorganic and organic amendments on the community of microorganisms in the microenvironments of maize demonstrated that the communities of microorganisms (including archaea, fungi, and bacteria) were considerably higher in soil treated with compost manure and different from those amended with higher amounts of synthetic fertilizers [42]. Furthermore, the addition of compost manure to the rhizosphere soil produced the highest levels of bacterial, archaeal, and fungal diversity and richness. This is in line with the results of Ma et al. [82], who demonstrated that organic fertilizers (T40, T100) enhanced the proportion of helpful microorganisms in the garlic rhizosphere and promoted plant growth and nutrient cycling. They also emphasized that the application of biological fertilizers boosted the intricacy of communications and cooccurrences among soil bacteria and archaea, fostering competitive relationships between bacterial populations, as illustrated through network analysis. Additionally, in contrast to chemical fertilizers, organic fertilizers raised the populations of soil microbiomes and nutrients (carbon and nitrogen), stabilize pH levels, and enhanced garlic yield.

Supporting these findings, Zhao et al. [128] show that using metagenomics sequencing, Arbuscular mycorrhizal fungi (AMF) introduction significantly shaped the structure, diversity, as well as versatility of the community of microorganisms within the soil derived from *Iris tectorum* rhizosphere. The community of microorganisms was significantly richer and more abundant in the treatment group that was inoculated with AMF.

Similarly, in an experiment to investigate the effect of biofertilizers on crop performance, Ajilogba et al. [8] reported that Bambara groundnut (*Vigna subterranean*) plants treated with rhizobacteria isolated from their rhizosphere produced up to twice as many seeds per plant compared to untreated controls. The *Bacillus spp*. enhanced nutrient availability and uptake by promoting nitrogen fixation, synthesizing growth-promoting compounds such as indo-3-acetic acid (IAA) and other phytohormones, as well as facilitating phosphate solubilization thereby resulting in improved plant growth and productivity under field conditions.

However, it is important to note that many microorganisms remain unculturable under standard laboratory conditions, which restricts our insight into the entire scope of microbial diversity. This is where shotgun sequencing comes into play [15, 74], as it allows researchers to profile both culturable and unculturable microbes, providing a holistic view of the rhizosphere’s microbiome [14, 16]. While previous studies have relied on microscopic observation and 16S rRNA sequencing to identify microorganisms from the rhizosphere crops, these approaches are solely meant for certain groups of microbes, and none of them can contribute a detailed overview of the community structure and the diversity of the total microbial population within the soil specimen. This highlights the need for more advanced techniques like shotgun sequencing to provide comprehensive information about the microbial diversity and the community structures within the soil samples [14, 16]. However, the consequences of chemical fertilizer as well as biofertilizer treatment on enriching the diversity of microorganisms and community structure of *A. ampeloprasum* rhizosphere particularly in a semi-arid environment are still not completely understood. Thus, the goal of this investigation is to profile the microbial diversity and rhizosphere-related community structure for *A. ampeloprasum* under different fertilization using a shotgun metagenomics sequencing.

We hypothesized that the integration of synthetic as well as biofertilizer would possibly alter the diversity as well as the structure of the rhizosphere community of microorganisms. We expect that this alteration will vary depending on the composition and the variety of fertilizer employed, and that these shifts in microbial diversity will influence *A. ampeloprasum’s* growth. To confirm this hypothesis and learn more about the relationship between various fertilization systems and the growth of *A. ampeloprasum* plant, we conducted a shotgun metagenomics sequencing to examine the influence of biofertilizer and chemical fertilizers on the taxonomic abundance, diversity, and structure of *A. ampeloprasum* rhizosphere microbiomes. We also sought to ascertain whether the rhizosphere’s diversity and structure of the microbial community varied amongst the fertilizer plots compared to the bulk soil, providing an understanding of the specific impacts of each treatment on the dynamics of the soil community of microorganisms.

## Experimental procedures and materials

### Research location and soil collection protocols

Samples of rhizosphere soil were sourced from Rosaly Farm (26°06'10.7"S, 27°34'41.8"E), located in the Gauteng Province of South Africa. The farm covers an area of 15,000 m^2^ and is divided into three plots (each plot is 20 m apart from the other). The first plot was treated with chemical fertilizer (G1), while the second plot was treated with biofertilizer (G2), and the third plot, called the bulk soil (G3), received no treatment. The sampling site is characterized by an average temperature of 22 °C, yearly rainfall of 794 mm, and pH values that are slightly alkaline, ranging from 7.21 to 7.33 (Table 1 and Supplementary Table S1). The sand content ranged from 77.50 to 79% with minimal variation and a moderate clay content. Samples of rhizosphere soil were obtained on day 57 after sowing during the bolting stage from *A. ampeloprasum* rhizosphere (15 mm depth) under different fertilization systems (chemical fertilizer and biofertilizer), along with uncultivated bulk soil were collected in four replicates each: L1, L2, L3, and L4 for G1; L9, L10, L11, and L12 for G2; and LB1, LB2, LB3, and LB4 for G3, producing 12 samples in total. To eliminate the risk of contamination by fertilizer impacts and root secretions, bulk soil was obtained from an uncultivated plot sited 20 m away from the fertilizer plots. Samples of soils collected were conveyed in an ice box into the laboratory and preserved for subsequent use. The samples were arranged separately, sifted, and later preserved in sealable containers at 4 °C.

**Table 1:** Properties of the soil in *A. ampeloprasum* rhizosphere and bulk soils

Physicochemical values	Samples ID		
	G1	G2	G3
pH	7.33 ± 7.34^a^	7.21 ± 0.03^a^	7.26 ± 0.03^a^
P (mg/kg)	208.75 ± 8.75^a^	230.25 ± 10.17^a^	222.00 ± 8.50^a^
K (mg/kg)	173.25 ± 0.49^a^	217 ± 0.86^a^	211.5 ± 0.65^a^
Ca (mg/kg)	942.25 ± 0.99^a^	927.75 ± 0.87^a^	897.75 ± 0.91^a^
Mg (mg/kg)	135.75 ± 0.87^a^	128.25 ± 0.23^a^	129.5 ± 0.26^a^
Na (mg/kg)	1.5 ± 0.05^a^	2 ± 0.10^a^	1.25 ± 0.10^a^
N-NO_3_ (mg/kg)	4.17 ± 1.14^a^	8.86 ± 1.13^b^	5.59 ± 0.81^b^
N-NH_4_ (mg/kg)	1.33 ± 0.10^a^	1.72 ± 0.12^a^	1.43 ± 0.16^a^
Total N (mg/kg)	417.50 ± 10.08^a^	479.50 ± 23.01^a^	414.75 ± 32.25^a^
Sand (%)	79.00 ± 0^a^	78.75 ± 1.03^a^	77.50 ± 0.65^a^
Silt (%)	8.25 ± 1.10^a^	8.25 ± 0.25^a^	7.75 ± 0.48^a^
Clay (%)	12.75 ± 1.10^a^	13.00 ± 1.08^a^	14.75 ± 0.75^a^
C (%)	0.53 ± 0.01^a^	0.60 ± 0.04^a^	0.52 ± 0.04^a^
S—value	6.28 ± 0.19^a^	6.26 ± 0.23^a^	6.11 ± 0.20^a^
Moisture content	6.28 ± 1.34^a^	6.07 ± 1.01^a^	5.15 ± 0.48^a^

Mean and standard errors (n = 4); means were significantly distinct (*p* < 0.05) concerning t-test. G1 = Rhizosphere soil from *A. ampeloprasum* crop from chemical fertilizer plot; G2 = Rhizosphere soil from *A. ampeloprasum* crop from biofertilizer plot; G3 = Bulk soil samples from uncultivated soil as control. Means whose letters are identical are not significantly different from one another

The farm (Plot A) holds a history of chemical fertilizer application, consisting of 150 g of calcium nitrate (Ca (NO_3_)_2_), 120 g of ammonium sulfate ((NH_4_)_2_SO_4_), 200 g of potassium nitrate (KNO_3_), 150 g of magnesium nitrate (Mg (NO_3_)_2_), 150 g of potassium sulfate (K_2_SO_4_) and 100 g of N: P: K (5:1:5) per square meter (g/m^2^) applied. Plot B has a record of using 0.56 g of terramax, a natural organic extract comprising a variety of plant growth-enhancing rhizobacteria (PGPRs); 0.82 liter of humesoil, an organic formulation blended with carefully selected organic materials, derived from plants and trees; and 0.494 ml of soluphos, a phosphate-solubilizing microbial inoculant in organic powder form that contains *Pseudomonas putida*, *Bacillus licheniformis* at 4.8 × 10^9^ CFU/g, and microbial food, being applied per square meter (/m^2^) as biofertilizers. Chemical fertilizers and biofertilizers were applied to assess their impacts on soil microbial composition, diversity and structure as well as plant growth in comparison to uncultivated bulk soils used as the controls.

### Evaluation of soil chemical and physical features

#### Soil parameter analysis of *A. ampeloprasum* rhizosphere and bulk soils

Each sample’s soil parameters were analyzed independently within 14 days of the sample collection. For physicochemical examination, 20 g samples of the soil were crushed, air dried, and well mixed, followed by sifting with a 2 mm screen mesh to separate solids and debris [39]. The size of the soil particles was analyzed with a hydrometer [86]. The soil’s pH was assessed through a pH meter with 1:2.5 soil in distilled water [17], and total nitrogen was determined as described by [78]. At pH 7.0 with ammonium acetate (1 M) technique, exchangeable sodium (Na), potassium (K), magnesium (Mg), and calcium (Ca) were evaluated following extraction. To measure the exchangeable potassium ion (K^+^), a flame photometer was employed, while exchangeable Ca^2+^ and Mg^2+^ were measured via an atomic absorption spectrophotometer (AAS) [106]. Using spectrophotometry, obtainable phosphorus (P) was evaluated, and organic carbon was estimated via the Walkley Black protocol [27]. Available NO_3_^−^ and soil exchangeable NH_4_^+^ were evaluated using the protocol by [52]. The silt, clay, and sand percentage proportions were calculated via methods outlined by Mozaffari et al. [87].

### Metagenomic DNA extraction and shotgun sequencing

Following the instructions on the DNeasy® PowerSoil Pro Kit (Qiagen, Germany), 20 g of soil samples from each plot were employed to mine the DNA. The mined DNA from every sample was then sequenced via the Illumina NovaSeq X Plus (PE 150) platform. Using a Covaris disruptor, DNA was fragmented to approximately 350 bp to prepare the library, after which end repair, PCR amplification, and adapter ligation were performed. AATI analysis was used to confirm the insert size of the library, and Q-PCR confirmed concentrations greater than 3 nM. Pooling and sequencing of verified quality libraries were carried out with the help of the PE150 advancement technique. Pre-processing of data was achieved through Fastp [23] to erase adapters and reads of low-quality, and Bowtie2 [72] was employed to get rid of host impurities. MEGAHIT [75] used the meta-large preset to compile the clean reads, and scafftigs were made by discarding scaffolds with “N” junctions [73].

Prediction of ORF was executed via MetaGeneMark [84], and sequences below 100 nt were discarded. CD-HIT was employed to discard excessive ORFs, leading to a non-redundant or unique gene database [26]. Mapping of clean data was carried out on this database through Bowtie2, and genes with read counts ≤ 2 were discarded for subsequent examination [125]. The abundance of genes was estimated according to the gene length and read counts [110]. DIAMOND (https://github.com/bbuchfink/diamond/) [19] aligned unigenes sequences to the Micro_NR database which comprises of sequences from viruses, archaea, bacteria, and fungi retrieved from NCBI’s NR database (https://www.ncbi.nlm.nih.gov/) and the alignment was carried out with the use of the blastp algorithm, with a parameter settings 1e-5 [67] as well as KEGG database (http://www.kegg.jp/kegg/) [63, 64] for functional annotation likewise PHI database (http://www.phi-base.org/index.jsp) for diseases incidence estimation.

Species identification was allocated through the LCA algorithm adopted for systematic taxonomy in the MEGAN program software (https://en.wikipedia.org/wiki/Lowest_common_ancestor) which was applied to determine the species-level annotation information of the sequence [48, 57, 76]. From the outputs of the LCA annotation and gene abundance table, the abundance of each sample in each taxonomy (kingdom, phylum, class, order, family, genus or species) and the respective gene abundance tables were obtained. The abundance of a species in a sample is the sum of the abundance of those genes annotated as that species [66].

Based on the abundance tables at each taxonomy level, a relative abundance summary, and an abundance clustering heatmap were generated (R ade4 package). Species variations among groups were sorted for by employing MetaGenomeSeq and LEfSe analysis. Permutation test between groups of each taxonomic level were conducted using MetaGenomeSeq analysis with a p-value. LEfSe analysis was carried out using LEfSe software (LDA score is 4 by default) [101]. Relative abundance was estimated through the R ade4 package [99] and illustrated with the use of Excel [122]. Diversity indices were estimated through the “vegan” R package [90], and ANOSIM was employed to estimate group variances.

Similarly, selection of species by gradient at species level was executed through the Random Forest analysis with the assistance of R pROC and randomForest packages [18] and a random forest model was constructed. Species that are of significance value were filtered out by MeanDecreaseGin and, MeanDecreaseAccuracy and then rotation estimation (predefined tenfold) is carried out for all the model, and the ROC curves were drawn.

### Statistical analyses

Physicochemical parameter differences were determined by comparing means via a one-way analysis of variance (ANOVA) and applying Tukey’s pairwise comparison test at the significance level (*p* < 0.05). The Pielou Chao1, Shannon, and evenness diversity indices were assessed for each sample, and the results were compared between habitats using the Kruskal–Wallis test with R package software.

A one-way analysis of similarities (ANOSIM) with 999 permutations was utilized to look for differences in the community composition between the sample groups, and a principal coordinate analysis (PCoA) based on an Euclidean distance matrix was utilized to show the beta diversity [111]. The distribution of the microbial communities among the fertilizer plots and bulk soil samples was demonstrated through a principal component analysis (PCA) built on a Euclidean distance matrix.

To evaluate the parameters of the environment that best decipher bacterial diversity and structure, we performed redundancy analysis (RDA), and for validation as well as a significance test, the Monte Carlo permutation test, making use of 999 random permutations, was employed. All the parameters of the environment itemized in Table 1 were integrated in the RDA as illustrative factors. The Bray–Curtis dissimilarities matrix based on principal coordinate analysis (PCOA) was used to demonstrate how the assessed phyla were distributed across the various sites. The RDA, PCoA as well and PCA graphs were represented via CANOCO (version 5.0). In the NCBI-SRA database, the quality-filtered sequences are openly found and accessible under accession numbers SRP537120, SRP537121, and SRP537188.

## Results

### Physicochemical parameters analysis of soil

The analysis of the soil’s physicochemical characteristics uncovered that the nitrate nitrogen (N-NO_3_) is significantly (*p* < 0.05) higher in soil samples from biofertilizer plots and the bulk than in the chemical fertilizer plot soil samples, while other physicochemical parameters did not differ significantly across the soil samples.

### Data sets for shotgun sequencing

After quality control evaluation, the average total sequence reads observed are 208,417,669.8 bp for the chemical fertilizer plot soil portions (G1) with a 65% of GC content, 215,776,116.5 bp for the biofertilizer plot soil portions (G2) with a 64% of GC content and 202,458,310.5 bp for bulk plot soil portions (G3) with a 64% of GC content. After quality filtering, sequence reads of acceptable quality with known protein functions amounted to 133,303,941 for chemical fertiliser plot samples (G1), 136,025,263 for the biofertilizer plot samples (G2), and 127,163,254 for bulk plot samples(G3) (Supplementary Table 2).

### Analysis of the structural composition of the microbial community

#### Distribution of major microbiome phyla across *A. ampeloprasum* rhizosphere soil under fertilization and bulk soil

The sequence data for the 12 *Allium ampeloprasum* rhizosphere soils under chemical fertilizer (G1), biofertilizer (G2) treatments, and bulk soil (G3) samples are provided in Table S2.

**Fig. 1:**
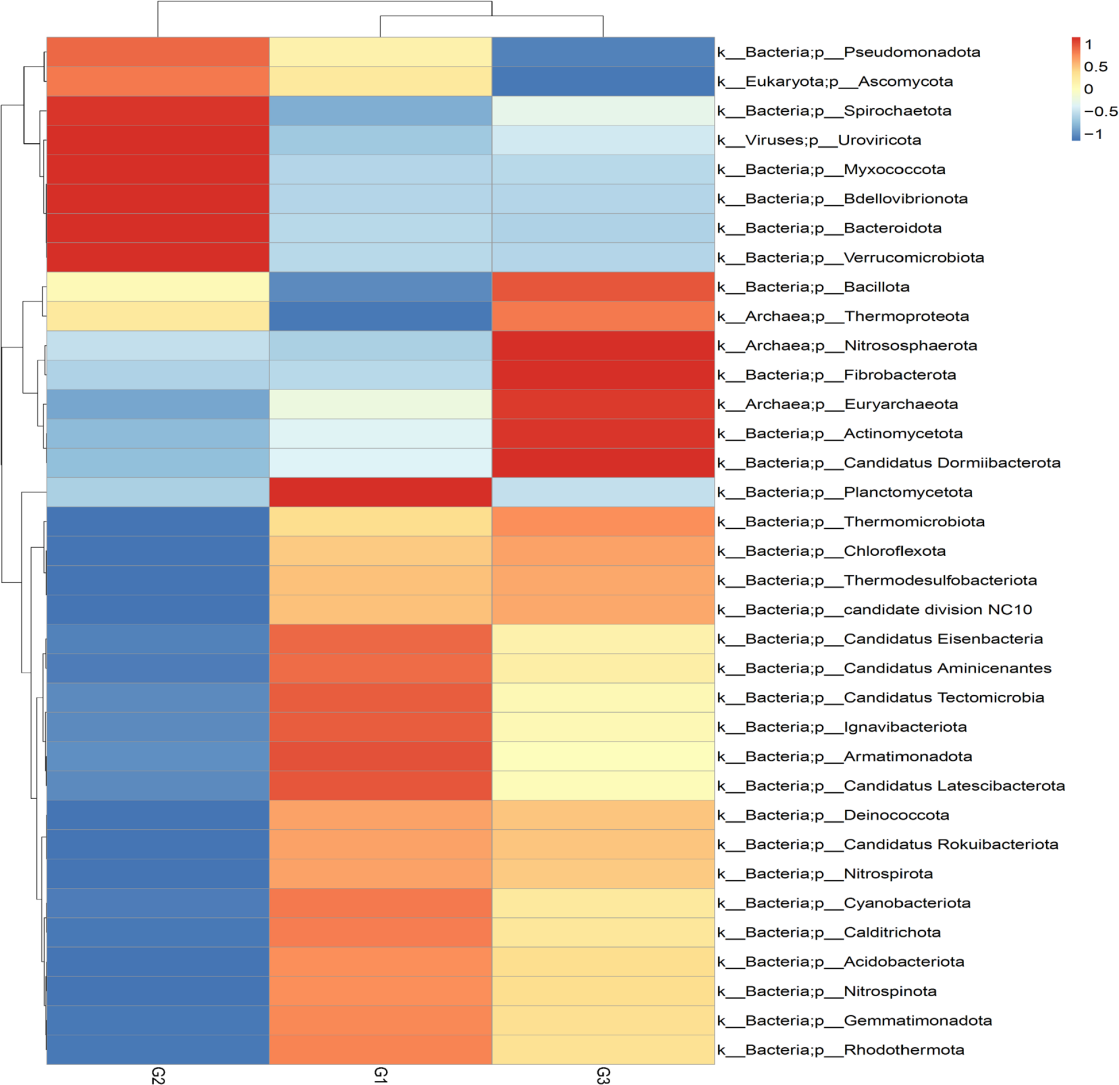
Heatmap showing the main phyla of the communities of the soil microbiome connected to the bulk soil samples and the rhizosphere of the *A. ampeloprasum* plant. The bars indicate the color intensity gradient employing the relative abundances with a z-score. G1 (Samples of rhizosphere soil from a chemical fertilizer plot); G2 (rhizosphere soil samples from biofertilizer plot); and G3 (Bulk soil samples from uncultivated soils)

**Fig. 2:**
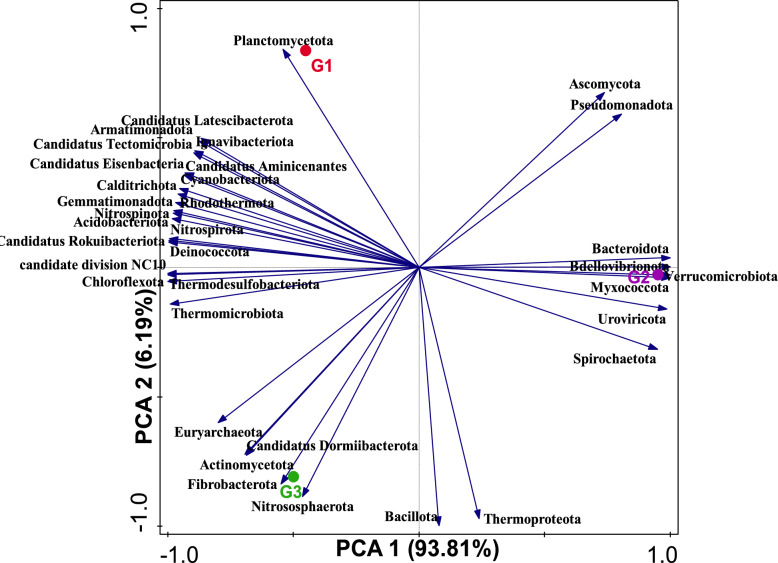
PCA graph illustrating the typical phyla of the soil microbial community. The strength of influence of microbial metagenomes is depicted by the vector arrow. Axis 1 (93.81%) and Axis 2 (6.19%) illustrate the variation through the Bray–Curtis dissimilarity matrix. G1 (Samples of rhizosphere soil from a chemical fertilizer plot); G2 (Rhizosphere soil samples from biofertilizer plot); and G3 (Bulk soil samples from uncultivated soils)

Analysis of the metagenomics data revealed a total of 35 phyla across the *A. ampeloprasum* rhizosphere acquired from G1, G2 and G3. The rhizosphere soil samples of *A. ampeloprasum* crop from the chemical fertilizer plot (G1) were dominated by Actinomycetota, Pseudomonadota, Acidobacteriota, Chloroflexota, Gemmatimonadota, Myxococcota, and Nitrososphaerota. However, rhizosphere soils of *A. ampeloprasum* crop from biofertilizer plot (G2) were dominated by Actinomycetota, Pseudomonadota, Acidobacteriota, Chloroflexota, Myxococcota, Bacteroidota, Verrucomicrobiota, Nitrososphaerota, and Gemmatimonadota. Moreover, samples from bulk soil reveal a relative microbial abundance of the phyla Actinomycetota, Pseudomonadota, Acidobacteriota, Chloroflexota, Myxococcota, Gemmatimonadota, and Nitrososphaerota (Fig. 1 and supplementary Table 3). The only fungal phylum identified, namely Ascomycota, was identified across the plots (Supplementary Table S3). A PCA plot with axes 1 and 2 explaning 93.81% as well as 6.19% of the differences respectively, was employed to illustrate the phyla distribution amongst the bulk soil samples as well as the *A. ampeloprasum* rhizosphere (Fig. 2). The PCA’s vector length reveals which phylum dominates each group of soil samples (i.e., the phylum with the lengthiest PCA vector length) and how dominant each group is. Using this as an indication, it demonstrates for instance that phyla Planctomycetota, Candidatus Eisenbacteria, Candidatus Aminicenantes, Candidatus Tectomicrobia, Ignavibacteriota, Armatimonadota, Candidatus Latescibacterota, Cyanobacteriota, Calditrichota, Acidobacteriota Nitrospinota, Gemmatimonadota and Rhodothermota and candidate division NC10 (unclassified) predominated chemical fertilizer plot (G1), phyla Spirochaetota, Uroviricota, Myxococcota, Bdellovibrionota, Bacteroidota, Ascomycota and Verrucomicrobiota predominated biofertilizer plot (G2) while Nitrososphaerota, Fibrobacterota, Euryarchaeota, Actinomycetota and Candidatus Dormiibacterota predominated the bulk soil samples. *A. ampeloprasum* rhizosphere soils (G1 & G2) and the bulk soil (G3) microbiomes are separated along the two principal components, meaning that there is a significant difference between these groups based on their microbial diversity or community composition structure.

**Fig. 3:**
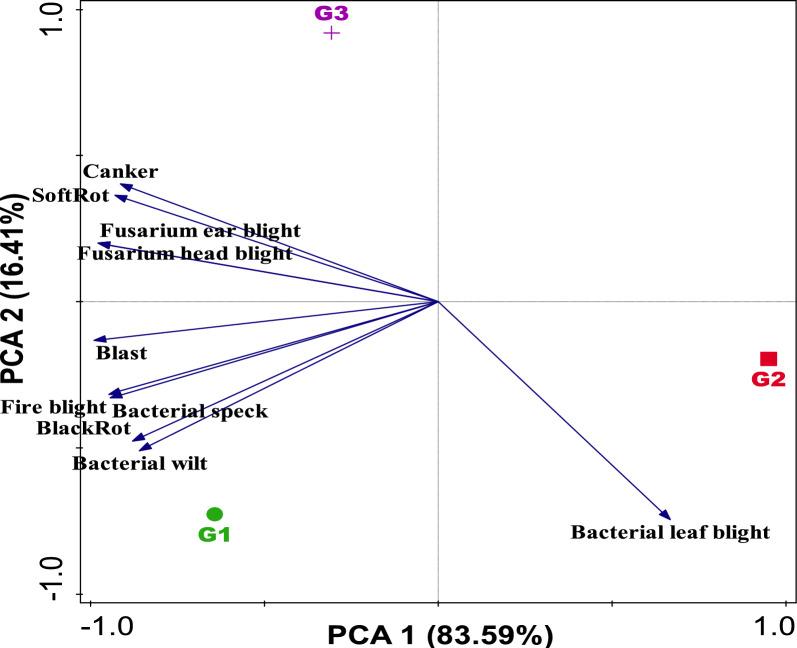
PCA graph illustrating the diseases incidence across the soil samples. The distribution of diseases incidence is depicted by the vector arrow. Axis 1 (83.59%) and Axis 2 (16.41%) illustrate the variation through the Bray–Curtis dissimilarity matrix. G1 (Samples of rhizosphere soil from a chemical fertilizer plot); G2 (Rhizosphere soil samples from biofertilizer plot); and G3 (Bulk soil samples from uncultivated soils)

Furthermore, the PCA plot (Fig. 3) revealed the distribution of disease incidence within the rhizosphere soils and bulk soil samples based on the level 1 of the PHI-coefficient (which corresponds to disease incidence), explaining a total variation of 83.59%. The direction and length of the vectors indicate the distribution of the diseases incidence as influenced by the soil samples. Using this as a guide, blast, fire blight, bacterial speck, black rot and bacterial wilt were prevalent in the chemical fertilizer-treated soil, followed by the bulk soil samples where canker, soft rot, fusarium ear blight, and fusarium head blight were prevalent. However, the biofertilizer-treated soil (G2) shows the lowest diseases incidence with only bacterial leaf blight as the dominant disease but the variation found across the sites is not significantly different (*P* > 0.05) across fertilization plots (Fig. 3 and Supplementary Table 6). Although the PCA revealed a relatively lower incidence of diseases in group G2 compared to groups G1 and G3, an ANOSIM of level 1 was performed to determine the dissimilarities in the pattern of disease incidence among the groups. The ANOSIM R statistic was 0.062, with a related p-value of 0.241. The R value depicts a low level of separation between the groups considering the incidence profiles of diseases.

#### Structural composition of the microbial community across *A. ampeloprasum* rhizosphere soil samples under fertilization and bulk soil

The relative abundance of Pseudomonadota (16.08%) and Bacteroidota (2.47%) in the biofertilizer plot (G2) at the phylum level were greater than Pseudomonadota (G1 = 15.27 and G3 = 14.02%) and Bacteroidota (G1 = 0.40% G3 = 0.35%) in the chemical fertilizer plot (G1) and bulk plot (G3) respectively. While Actinobacteria (43.95%) and Chloroflexota (8.14%) from bulk soil samples were higher than Actinobacteria (42.67% and 42.32%) and Chloroflexota (8.02% and 6.93%) in the chemical fertilizer plot (G1) and bulk soil plot (G3) respectively (Fig. 1 and Supplementary Table S3).

**Fig. 4:**
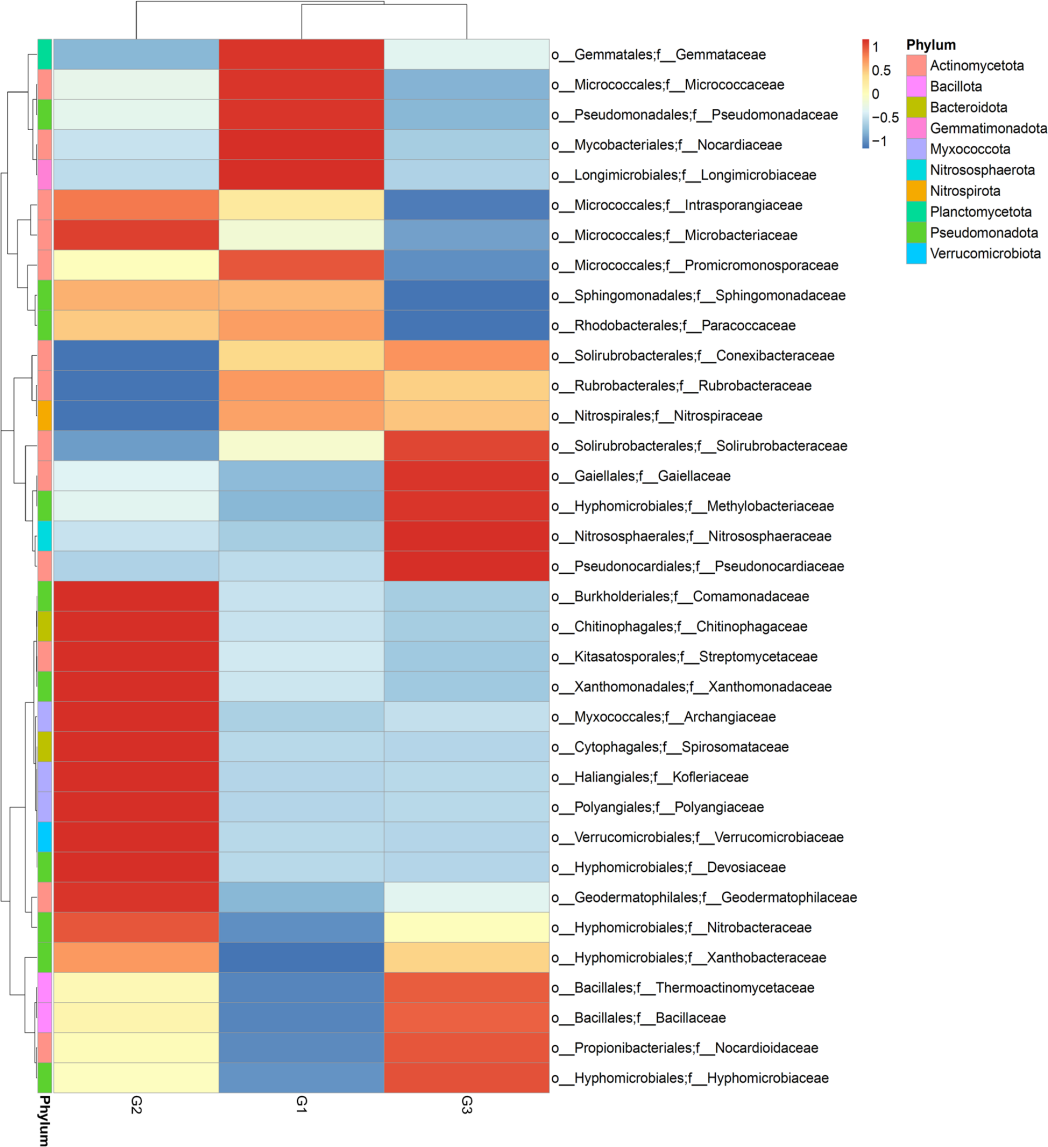
Heatmap showing the main families of soil microbial communities associated with the bulk soil samples and the rhizosphere of the *A. ampeloprasum* plant. G1 (Samples of rhizosphere soil samples from a chemical fertilizer plot); G2 (Rhizosphere soil samples from biofertilizer plot); and G3 (Bulk soil samples from uncultivated soils)

**Fig. 5:**
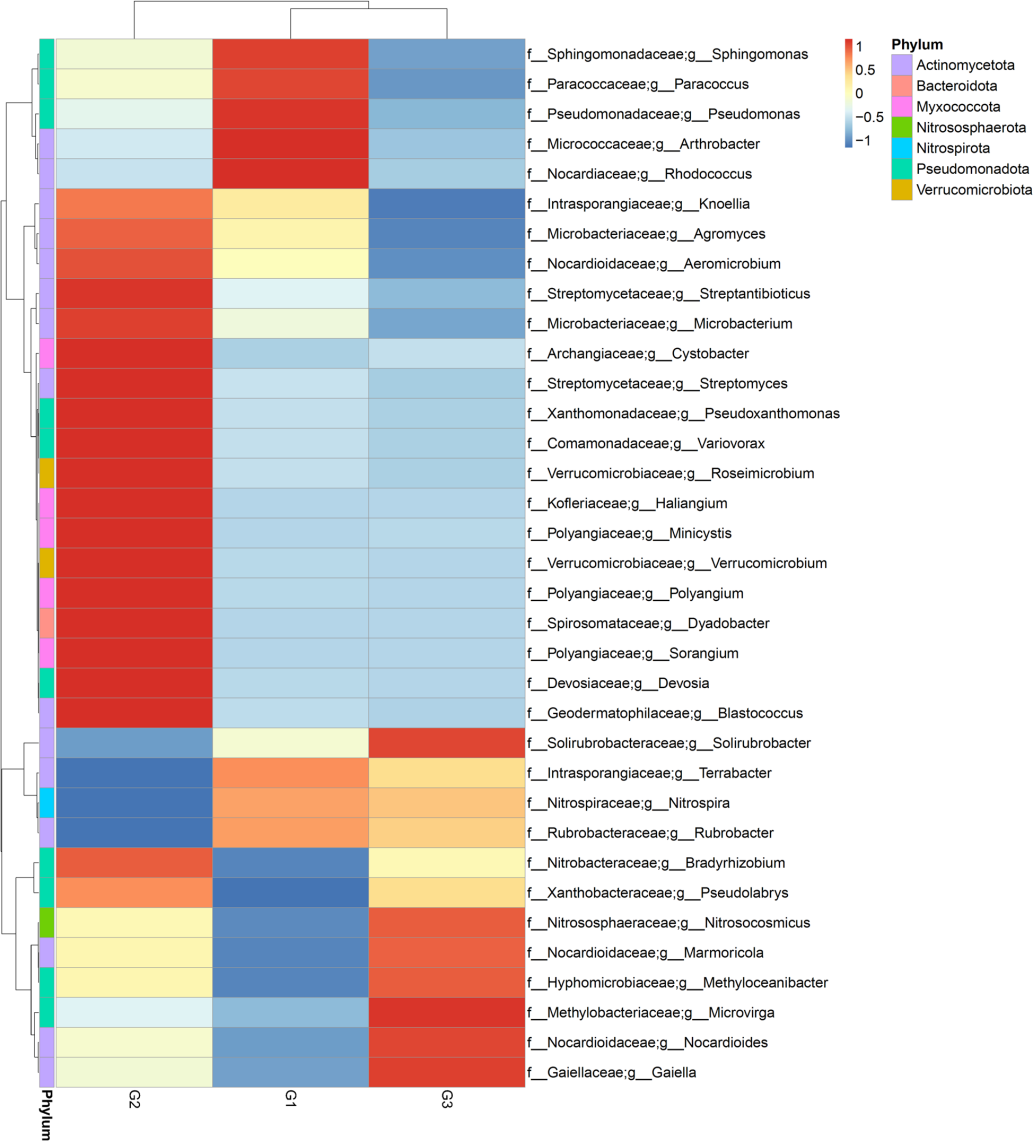
Heatmap showing the main genera of the community of the soil microbiome related to the bulk soil samples and the rhizosphere of the *A. ampeloprasum* plant. G1 (Samples of rhizosphere soil from a chemical fertilizer plot); G2 (Rhizosphere soil samples from biofertilizer plot); and G3 (Bulk soil samples from uncultivated soils)

The relative abundances of Spirosomataceae (1.78%), Verrucomicrobiaceae (1.62%), Streptomycetaceae (2.26%), Kofleriaceae (0.93%), Polyangiaceae (0.95%), Intrasporangiaceae (2.27%), Microbacteriaceae (2.09%), Comamonadaceae (0.97%), Xanthomonadaceae (0.83%), Archangiaceae (0.60%), Geodermatophilaceae (1.05%), Nitrobacteraceae (1.10%), Devosiaceae (0.33%), and Chitinophagaceae (0.20%) in soil samples from biofertilizer plot (G2) at the family level were greater than those of Spirosomataceae (0.01% and 0.01%), Verrucomicrobiaceae (0.05% and 0.03%), Streptomycetaceae (0.88% and 0.67%), Kofleriaceae (0.02% and 0.02%), Polyangiaceae (0.07% and 0.07%), Intrasporangiaceae (2.25% and 2.18%), Microbacteriaceae (1.39% and 0.94%), Comamonadaceae (0.29% and 0.21%), Xanthomonadaceae (0.21% and 0.13%), Archangiaceae (0.15% and 0.19%), Geodermatophilaceae (0.93% and 0.96%), Nitrobacteraceae (0.87% and 0.99%), Devosiaceae (0.04% and 0.03%), and Chitinophagaceae (0.09% and 0.08%) in soil samples from chemical fertilizer plot (G1) and bulk soil plot (G3) respectively. On the other hand, the relative abundances of Nocardioidaceae (14.58%), Solirubrobacteraceae (3.18%), and Gaiellaceae (2.05%) from bulk soil samples were higher than Nocardioidaceae (12.97% and 13.81%), Solirubrobacteraceae (2.92% and 2.75%) and Gaiellaceae (1.79% and 1.84%) in soil samples from chemical fertilizer plot (G1) and biofertilizer plot (G2) respectively (Fig. 4 and Supplementary Table S4). At the genus level, *Dyadobacter* (1.68%), *Verrucomicrobium* (1.12%), *Streptomyces* (1.63%), *Pseudoxanthomonas* (0.59%) and *Variovorax* (0.58%) were more abundant in the soil samples from biofertilizer plots than *Dyadobacte*r (0.00% and 0.00%), *Verrucomicrobium* (0.01% and 0.00%), *Streptomyces* (0.67% and 0.57%), *Pseudoxanthomonas* (0.07% and 0.03%) and *Variovorax* (0.08% and 0.05%). On the other hand, *Nocardioides* (12.72%), *Solirubrobacter* (2.59%), *Gaiella* (1.59%) and *Nitrosocosmicus* (0.86%) relative abundances in soil samples from bulk plot (G3) were higher than *Nocardioides* (11.39% and 11.94%), *Solirubrobacter* (2.41% and 2.28%), *Gaiella* (1.40% and 1.47%) and *Nitrosocosmicus* (0.66% and 0.77%) in the soil samples from chemical fertilizer plot (G1) and biofertilizer plot (G2) respectively (Fig. 5 and Supplementary Table S5).

#### Diversity estimation of microbial populations obtained from *A. ampeloprasum* rhizosphere soils and bulk soils

**Fig. 6:**
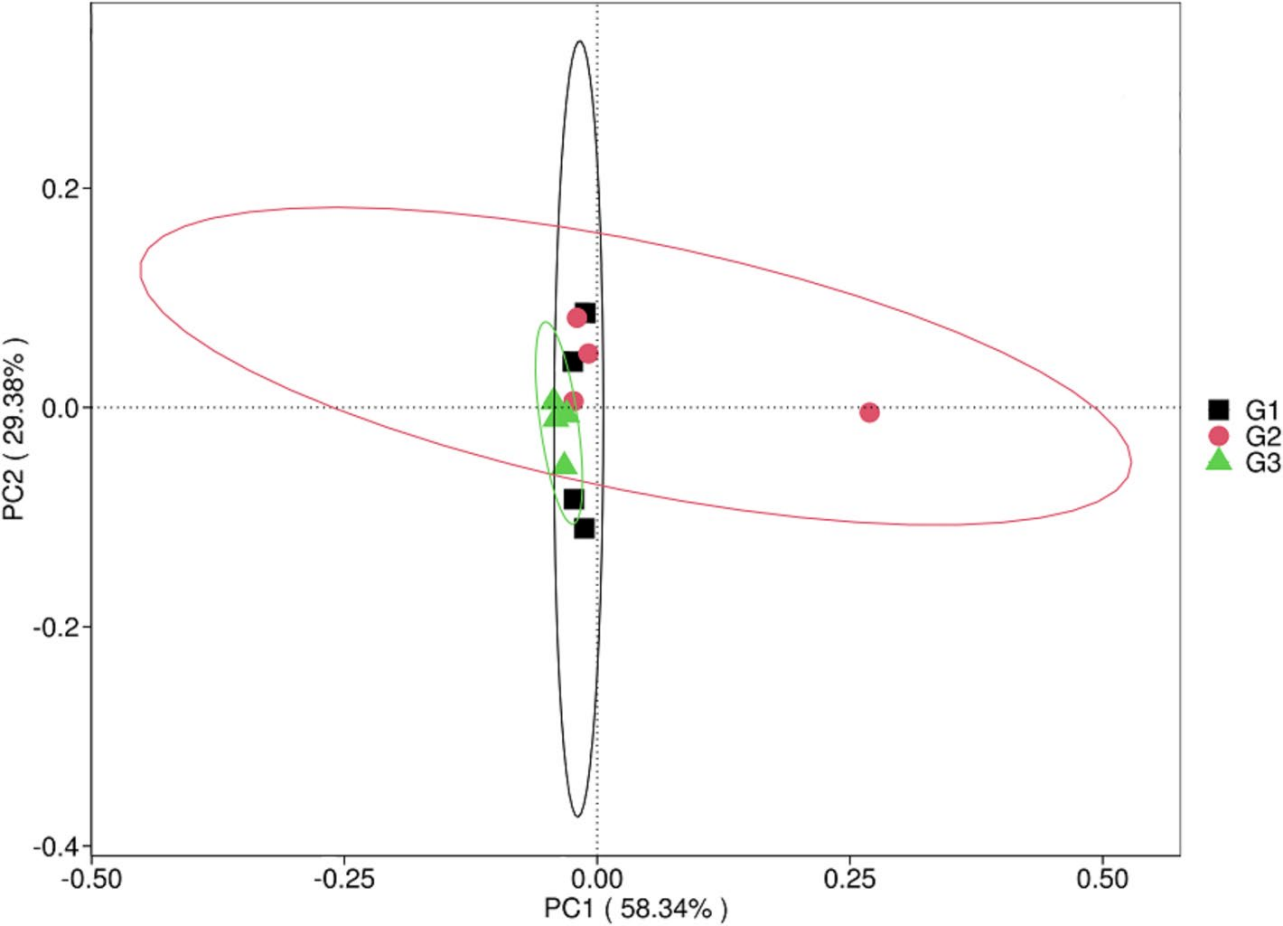
Graph showing the PCOA plot at the genus level of the soils’ community of microorganisms based on Bray–Curtis dissimilarities.  G1 (Samples of rhizosphere soil from a chemical fertilizer plot)  G2 (Rhizosphere soil samples from biofertilizer plot)  G3 (Bulk soil samples from uncultivated soils)

The Chao 1, Shannon, and evenness indices (alpha diversity indicators) at the genus, family, and phylum levels fell into the same range. For each sample (Table 2), there was no significant variation (Kruskal–Wallis *p* > 0.05). Additionally, the beta diversity study utilizing one-way ANOSIM did not reveal any significant difference (*p* = 0.19). The PCoA plot (Fig. 6) showed that the beta diversity analysis’s R value of 0.21 suggested a moderate connection between the samples. There was no clear clustering observed within the environments under investigation, according to the principal coordinate analysis that was performed to estimate and analyze the samples.

**Table 2:** Distribution of the means of the major microbiome phyla, families, and genera across the sites using the Chao1 and Shannon indices

Levels	Alpha diversity Indices	G1	G2	G3	*p*-value
Phylum	Chao 1	171.75	174.58	175.9	0.53
	Evenness	0.720	0.721	0.716	
	Shannon	2.57	2.59	2.57	
Family	Chao 1	750.55	770.68	759.77	0.16
	Evenness	0.908	0.904	0.886	
	Shannon	5.0	4.8	4.8	
Genus	Chao 1	2774.22	2835.00	2776.82	0.19
	Evenness	0.913	0.914	0.893	
	Shannon	5.80	5.68	5.61

### Influence of environmental factors on microbial communities

**Fig. 7:**
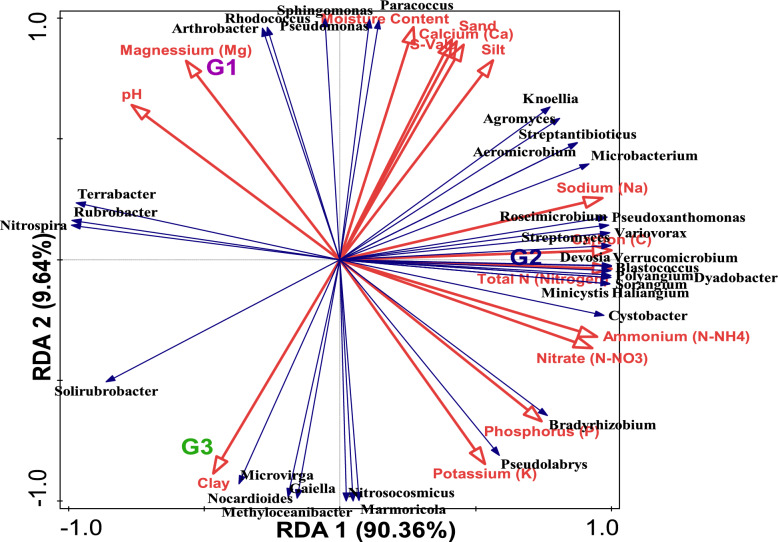
Redundancy analysis (RDA) showing the relationship between the microbial genera and major soil physicochemical parameters of the samples

**Fig. 8:**
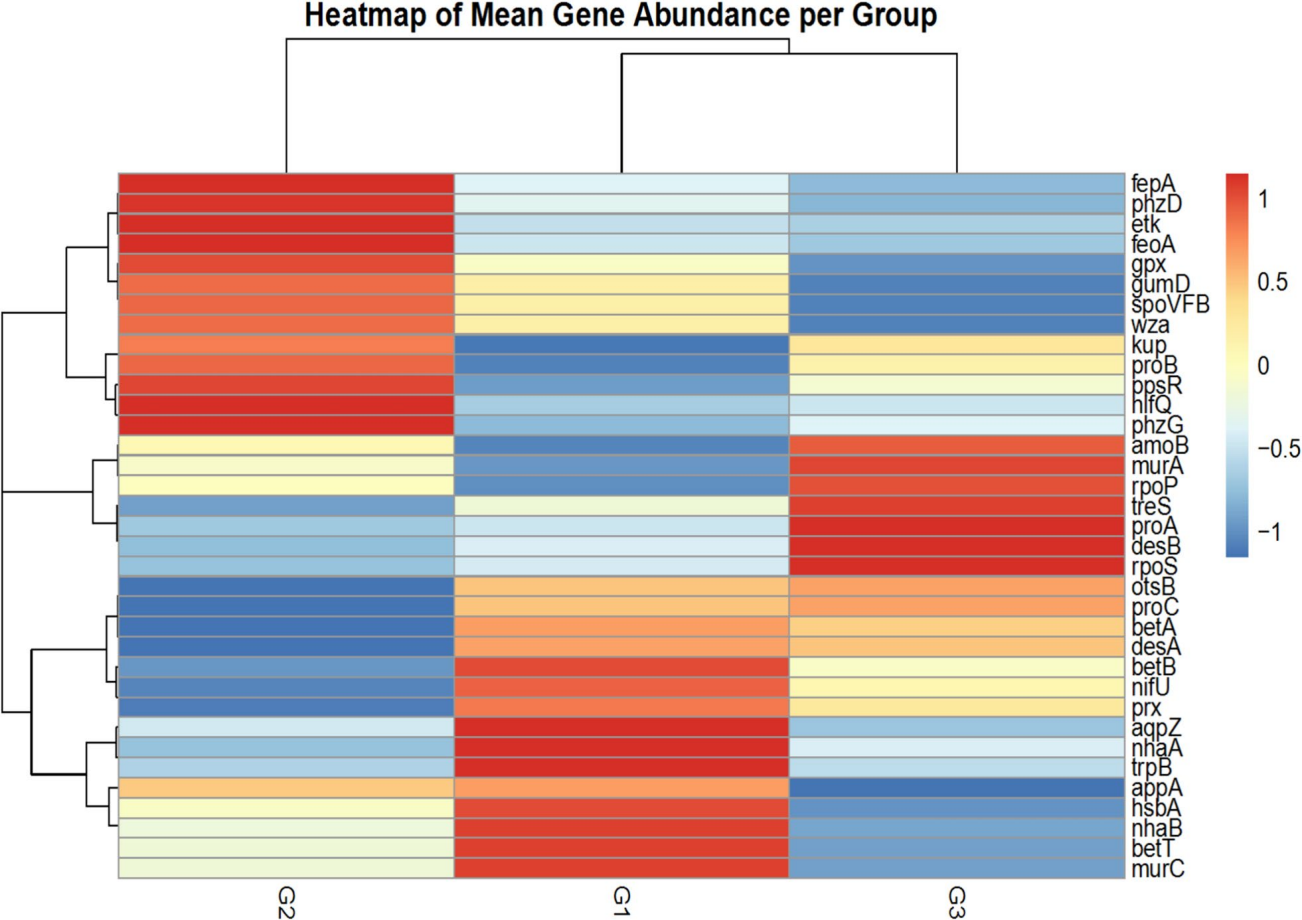
Heatmap showing the abundance of functional genes related to the bulk soil samples and the rhizosphere of *A. ampeloprasum* plants

**Fig. 9:**
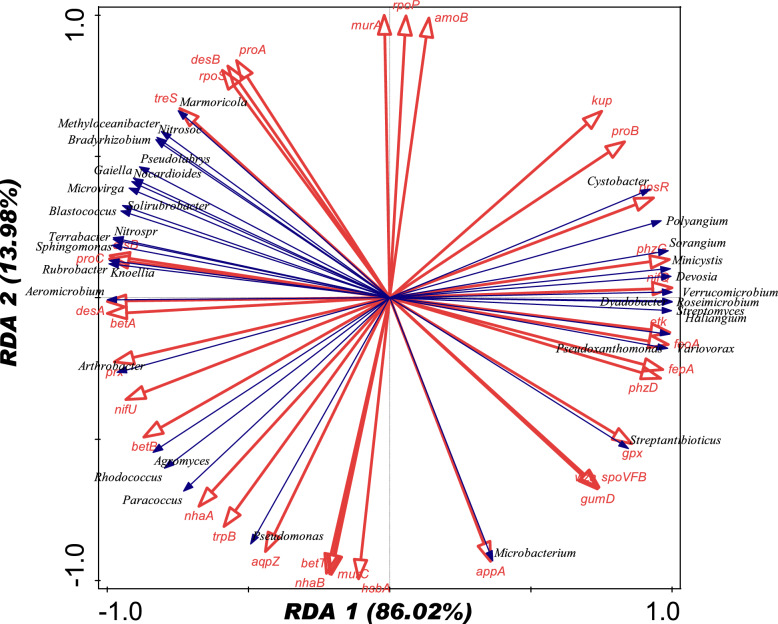
Redundancy analysis exploring the relationships between soil microbial genera and functional gene profiles across the soil samples

The redundancy assessment was employed to assess the relationship between the relative abundances within microbiome genera and the soil physicochemical characteristics that were considered (Table 1). For the RDA plot (Fig. 7), all soil parameters listed in Table 1 were utilized. Results from the RDA demonstrated that, with an RDA pseudoconical correlation of 1.0, the physicochemical characteristics of the soil most likely affect the makeup of microbial genus communities. The vector length of magnesium (Mg) and pH was positively correlated with *Arthrobacter, Arthrobacter, Terrabacter, Rubrobacter, Sphingomonas* and *Pseudomonas* and the length of the vector for nitrate (N-NO_3_), carbon (C), ammonium (N-NH_4_) and total nitrogen (total-N) was found to be positively correlated with *Roseimicrobium, Pseudoxanthomonas, Streptomyces, Variovorax, Devosia, Verrucomicrobium, Blastococcus, Polyangium, Dyadobacter, Sorangium, Minicystis, Haliangium* and *Cystobacter*. Moreover, there was a positive correlation of the clay’s vector length with *Solirubrobacter, Microvirga, Nocardioides,* and *Methyloceanibacter* abundances (Fig. 7). The variables of the environment that clearly depict the variances in the microorganism structure of the soil samples were identified by applying the Monte Carlo permutation test with 999 random permutations and forward selection of environmental parameters. Total nitrogen (Total N) significantly contributed 90.2% of the total variation (p-value = 0.17), as shown in Table 3 (Figs. 8 and 9).

**Table 3:** Factors of the environment that best describe the diversity of microorganisms

Soil factors	Explains (%)	Contribution (%)	pseudo-F	*P*
Total N	90.20	90.20	9.2	0.17
C	9.80	9.80	< 0.1	1.00

#### Distribution of microbial functional traits supporting plant resilience and growth across the rhizosphere soil of *A. ampeloprasum* and uncultivated bulk soil samples

**Fig. 10:**
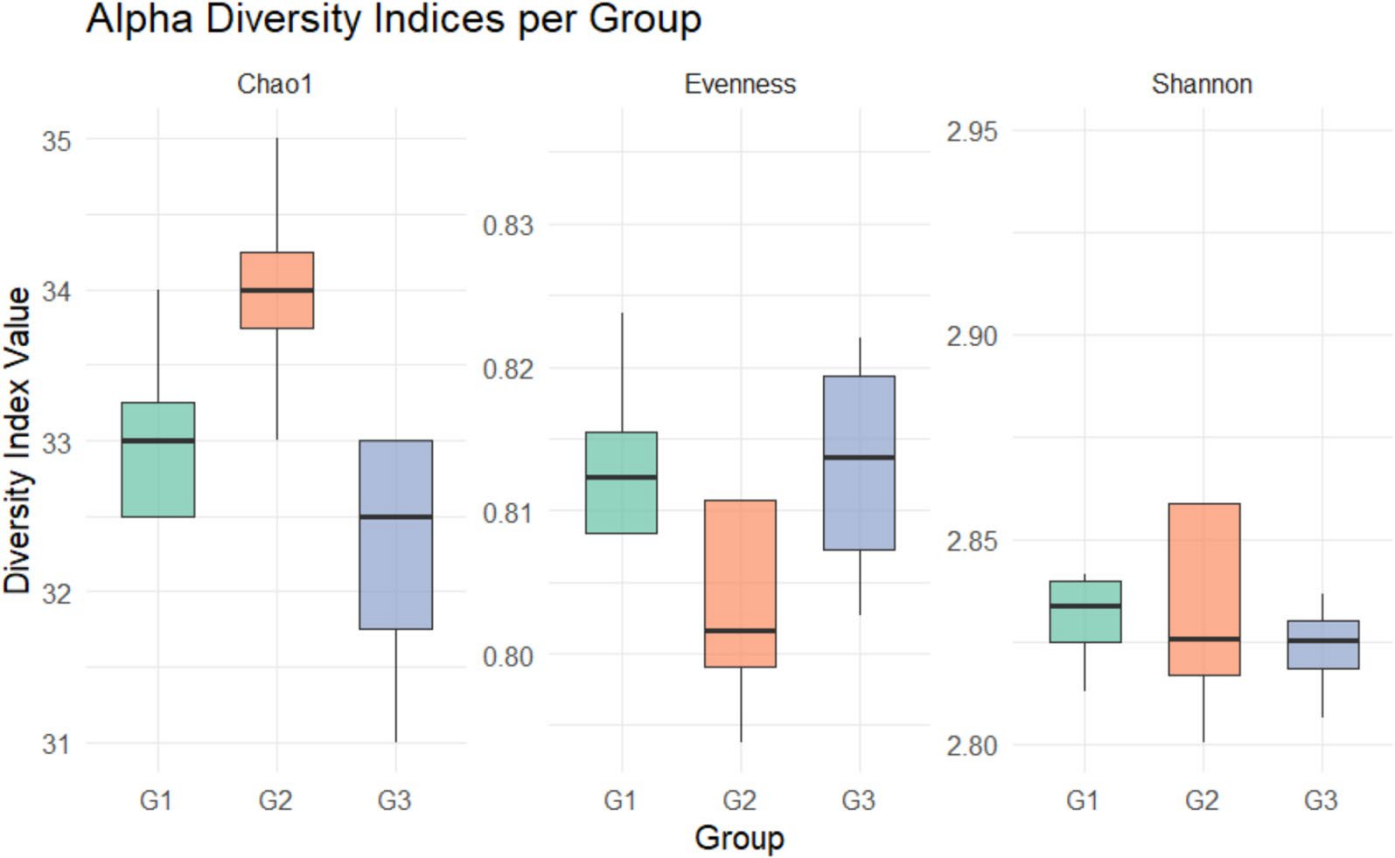
Alpha diversity indices of the functional genes across sample plots

Metagenomic analyses of *A. ampeloprasum* rhizosphere and bulk soil samples revealed that they are rich in microbial functional traits related to stress tolerance, nutrient acquisition, plant growth promotion, cell structure and biofilm formation (Fig. 8 and Supplementary Table 7). These genes were identified from all the soil samples and highlight the potential of microbial community’s functions in promoting plant growth and wellbeing. The chemical fertilizer plot (G1) is characterized by high abundance of functional genes such as aqpZ, betA, betB, betT, desA, murC, nhaA, nhaB, nifU, prx while the biofertilizer plot (G2) is characterized by a high prevalence of microbial gene like gpx, gumD, kup, nifQ, proB, phzG, phzD, fepA, wza and ppsr. In the same vein, the bulk soil samples were enriched with genes like desB, murA, otsB, proA, proC, amoB, and rpoS. The gene abundance of most of the genes under consideration do not vary significantly between the soil samples. Nevertheless, there were some genes, namely, gpx and prx, were significantly different between the soil samples under treatment (Fig. 8 and Supplementary Table 7). The results from the permutational multivariat analysis of variance (PERMANOVA) indicated modest variations in microbial functional traits profiles between the *A.ampeloprasum* rhizosphere soil and bulk soil samples (Supplementary Table S8). The alpha diversity indices (Chao1, evenness and shannon) (Fig. 10 and Supplementary Table 9) indicate that biofertilizer plot (G2) is inclined to have high richness and diversity values. Nonetheless, the evenness is comparable among groups, and this shows similar uniformity in the abundance distributions of species.

## Discussion

Conventional soil management practices in agricultural production, particularly those dependent on chemical fertilizers, have been proven to impair soil health and sabotage the diversity of soil microbial communities, thereby undermining long-term agricultural sustainability. In light of this, biofertilizer application is increasingly being embraced as a sustainable alternative to mitigate the detrimental effects of chemical fertilizers and promote agricultural sustainability.

Specifically, considerable efforts have been made with the application of shotgun metagenomics sequencing techniques to extensively profile the diversity and structure of the community of micro organisms in the bulk and *A. ampeloprasum* rhizosphere soils treated with biofertilizers (BF) and chemical fertilizers (CF), as soil microbial community and diversity are essential markers of soil health [115].

Our metagenome analysis revealed that the phyla Acidobacteriota, Gemmatimonadota, Planctomycetota, and Nitrospirota were dominant in the chemical fertilizer plot (G1), whereas Pseudomonadota, Myxococcota, Bacteroidota, and Verrucomicrobiota were the main bacterial phyla that were predominant and distinct in the biofertilizer plot (G2), and Actinomycetota, Chloroflexota, Nitrososphaerota, Bacillota, and Thermomicrobiota were dominant in the bulk soil samples (G3) (Fig. 1 and Supplementary Table S3). The majority of the microbial phyla identified have been previously documented, and their immense roles in improving soil health and functioning, as well as promoting the development and growth of host crops such as barley [41] and maize [10, 34] have been recognized. Differences in the abundance of bacterial phyla across the fertilization plots particularly those with high prevalence in G2, indicate that biofertlizers may serve as effective and sustainable options for enhancing soil microbial diversity and richness. This approach supports improves crop yield, suppresses plant diseases, and promotes overall biological health of the soil in an environmentally friendly manner [77]. At the genus level, *Pseudomonas, Sphingomonas, Arthrobacter, Nitrospira*, and *Rubrobacter* dominated the G1 plot, while *Dyadobacter, Verrucomicrobium, Streptomyces, Haliangium, Pseudoxanthomonas*, and *Variovorax* were dominant in the G2 plot. However, *Nocardioides, Solirubrobacter, Gaiella,* and *Nitrosocosmicus* were dominant in G3 plot (Fig. 5 and Supplementary Table S5). The majority of the identified bacterial genera have earlier been proven to positively enhance plant growth as phosphate solubilizers (*Arthrobacter* and *Streptomyces*) [112, 114], atmospheric nitrogen fixers and biocontrol agents (Pseudomonas, *Sphingomonas,* and *Streptomyces*) [61, 119, 121], indole-3-acetic producers (IAA) (*Sphingomonas*) [79]. Some have also been reported to exhibit ACC deaminase activity (*Variovorax and Sphingomonas*) [2, 44], are siderophore producers (*Pseudomonas, Arthrobacter*, and *Streptomyces*) [83], and antibiotic producers (*Streptomyces*) [98].

The majority of the microbial phyla found to be abundant in the biofertilizer plot (G2) have previously been reported to exhibit drought resistance in arid or semi-arid conditions, including *Pseudomonadota,* [47], *Verrucomicrobiota* (Guevara-Hernandez et al., 2024), Myxococcota [59], and *Bacteroidota* [105]; It is well known that these bacteria improve the efficiency of water uptake in soils under drought stress. This is attributed to their biofilms formation and root colonization, which improve the rhizosphere’s accessibility to nutrients like phosphorus and nitrogen [65]. These microbiomes also assist plants sustain cellular water balance amid drought conditions by producing osmoprotectants, which stabilize membranes and proteins and protect plant cells from oxidative stress and dehydration [96]. However, collaborative interactions between various microbial species, can help plants cope with drought. Fungi and bacteria, for example, can form symbiotic relationships in which fungi improve nutrient uptake and water absorption while the bacteria fix nitrogen [70].

The application of the shotgun metagenomics sequencing approach provided a comprehensive insight into the dominant archaeal and fungal taxa present in the rhizosphere of *Allium ampeloprasum* under different fertilization systems and in bulk soil*.* Three major archaeal phyla *Thermoproteota, Nitrososphaerota*, and *Euryarchaeota* were identified in our metagenome results (Fig. 1 and Supplementary Table S3). These archaeal phyla have previously been recognized as a components of the microbiome present in maize (*Zea mays*) rhizosphere of [34, 42] and endophytes of *Allium spp.* [56]. Additionally, Ascomycota is the only fungal phylum found in this study, and it was identified to be dominant in biofertilizer-treated plot (G2) (Fig. 1 and Supplementary Table S3). Our findings concur with those of previous investigations, which also reported this phylum in the maize rhizosphere [34] and also *Atractylodes lancea* [22, 25]. Numerous studies have reported that the majority of the well-represented group of fungi related with the microenvironments of plants is Ascomycota [45, 46, 60]. The diversity of this fungal phylum (Ascomycota) varied significantly across the fertilization systems, and was relatively low in chemical fertilizer-treated soil (G1). This may be a result of chemical fertilizers which might have caused an imbalance in soil nutrients [95], salinity problems [50], or soil acidification [85]. The cumulative results of these effects may eventually decrease the need for symbiotic processes and suppress mycorrhizal development [97], which can lower fungal diversity, and thus directly or indirectly limit fungal growth and colonization [126].

Moreover, the rhizosphere soils from CF plot (G1) and the bulk soils (G3) all have low content of nitrates, potassium, phosphorus, organic carbon and potassium (Table 1) which could explain why there were few or no phyla eukaryote. This was affirmed by Chen, Xu, et al. [25], who demonstrated that fungal diversity and community structure are severely impaired in soil with reduced organic carbon thereby acting as a significant constraint to the functioning of the fungal component of soil ecosystems and Yang, Sun, et al. [120] who indicated that decrease in the abundance and functional role of fungal species are significantly tied to nutrients-deficient soils. Specifically, the lack of essential nutrients like nitrogen and potassium causes the shifts in the abundance of fungal diversity (including Ascomycota) and their functional potentials. Similarly, Fu and He [51] also revealed that the addition of mineral fertilizers over a lengthy period led to decreased fungal diversity as well as an increase in phytopathogens in comparison to organic fertilizers. Moreover, a decrease in organic carbon deprives the fungi, especially Ascomycota, of the energy and carbon resources they require, making them less abundant and diverse in the soil. The Scarcity of organic carbon limits fungal metabolism and fungal community complexity resulting in low fungal communities [22, 25, 123].

Similarly, the high abundance of microbial communities in the biofertilizer-treated plot (G2) can be attributed to their diverse functional capabilities, including fixing atmospheric nitrogen, solubilizing and mobilizing nutrients, producing plant growth promoting compounds (such as phytohormones and siderophores), improving soil physicochemical properties, producing root exudates, enhancing nutrient cycling, colonizing and persisting in the soil, and modulating microbial communities towards beneficial taxa [44, 113]. Our findings align with those of Wang et al. [113] and Enebe and Babalola [42], who demonstrated that the application of biofertilizer enhanced the microbial community structure in the maize rhizosphere, contributing to improved plant growth and development. In other similar studies where biofertilizers were combined with animal manure, they were found to increase the bacterial diversity and community structure in the rhizosphere of *Phoebe bournei* [117], lettuce [35] and maize [113].

Additionally, the higher diversity of microorganisms in *A. ampeloprasum* rhizosphere soils from biofertilizer plots (G2) could be related to the plants’ release of various root exudates from plants due to application of biofertilizer to the soil [36]. It has been well established that the exudates of roots released by parent plants regulate and establish relationships that exist between soil microorganisms and plant roots [6, 129]. These exudates aid in the decomposition of the organic substrate and provide rhizosphere microbes with a carbon source needed to engage in a favorable symbiotic or mutualistic associations with other beneficial microbes or partake in a more advantageous root inhabitation [81, 104]. This kind of underlying interaction frequently modifies the soil’s characteristics and establishes the community of rhizosphere microbial diversity and structure [102].

However, the analysis of our metagenome uncovered functional genes composition which largely comprises stress tolerance genes such as aqpZ and otsB (osmotic stress); betA, betB, and betT (osmotic and salt stress); desA desB (temperature and oxidative stress); gpx and prx (oxidative stress); nhaA and nhaB (salt and alkaline stress); proA, proB, and proC (osmoprotectant); and rpoS, treS, hsbA and spoVFB (general stress response) (Fig. 8 and Supplementary Table 7). Genes related to nutrients acquisition and metabolism functions which include nifQ and nifU (nitrogen fixation accessory proteins in nitrogen fixation); amoB (nitrification); fepA, kup, and feoA (siderophore, iron uptake and acquisition); appA (phosphorus solubilization); and phzD and phzG (micronutrient acquisition and microbial antagonism) are also well represented. However, the genes murA, murC, wza, etk, gumD, ppsR, rpoP, and trpB which are crucial for the promotion of plant growth, microbial cell wall synthesis, and biofilm formation, were also represented. Most of these genes have earlier been identified in rhizosphere microbial communities of tomato [3], maize [92], cereal crops [53], sunflower [4] as well as termite mound soil [40]. These genes generally support bacterial survival, stress resistance and metabolic functions which are all critical for establishing and maintaining beneficial relationships with plants to improve soil heal, quality and growth.

The observed relationship between microbial functional capacity and microbial diversity means that shifts in microbial community composition and structure can modulate the soil stability, ecosystem functions, fertility, and health under different fertilization systems.

Different communities of rhizosphere microorganisms (fungi, bacteria, and archaea) dominated each site, as indicated by the PCA graph, accounting for 93.81% of the variance observed in the soils from bulk and rhizosphere samples (Fig. 2). The arrows from the vector illustrate which phyla are most affected in the distribution, and the positions of each microbial group reveal the composition of sequences linked to their corresponding phyla. From this perspective, it is feasible to determine which group of microorganisms is more prevalent in each sample collection location relative to the other soil samples (Fig. 1).

Similarly, the PCA plot (Fig. 3) which accounted for 83.59% of the total variation observed in the disease incidence across the soil samples, shows which soil samples exhibited the most diseases incidence. As shown by the vector arrows in the PCA graph, the analysis revealed that the chemical fertilizer-treated soil (G1) exhibited the highest incidence of diseases, followed by the bulk soil sample (G3), while the biofertilizer-treated soil (G2) samples show the lowest rate of disease incidence (Fig. 3 and Supplementary Table 5).

The lowest of disease incidence exhibited by the biofertilizer-treated soil (G2) could be linked to the combined effects of beneficial species of microbes belonging to phyla Actinomycetota, Pseudomonadota, Baccillota, and Myxococcota. These bacteria do not only directly suppress soil-borne pathogens by the production of antibiotics, disease resistant genes, and competitive exclusion but also stimulate plant defenses and improve soil microbial structure, which fosters a disease-suppressive environment when compared with chemical fertilizer-treated soil (G1) and bulk soil samples (G3), where highest abundance of disease incidence was shown.

This finding agrees with Adedayo et al. [3], Das et al. [29], Lopes et al. [80], Yang, Liu, et al. (2025) where many of the dominant phyla have been reported to mitigate the effects of diseases pathogens by the production of disease resistant genes, siderophore production, antibiotic resistance and limiting pathogen growth. The abundance and activity of these phyla are often enhanced by biofertilizer application, which leads to a balanced and resilient microbial community capable of suppressing soil-borne diseases more efficiently than chemical fertilizers.

Equally, in order to validate the connection between the microbial community structure, as defined at the genus level, and the functional gene composition in the soil samples, redundancy Analysis (RDA) was utilized. In this analysis, it was possible to investigate the key microbial genera associated with specific functional genes in the soil samples. The RDA plot (Fig. 9) demonstrated strong associations between functional genes and microbial genera which amount to a total variation of 86.02%. Using this as a guide, the gene ppsR is known for its involvement in photosynthesis or stress response and correlates with *Cystobacter*. The gene phzC plays a crucial role in micronutrient solubilization and microbial antagonism, while nifQ is involved in the assembly and functioning of the nitrogenase; both correlate with *Sorangium*, *Devosia*, and *Roseimicrobium*. Similarly, gene appA is associated with nutrient acquisition, and gpx is important for oxidative stress tolerance; these genes correlate with *Microbacterium* and *Streptantibioticus* respectively. Meanwhile, the osmotic stress tolerance gene aqpz, the osmotic and stress tolerance gene betA, and the stress tolerance gene treS, as well as the antioxidant enzyme prx, which protects the plant against oxidative damage, are associated with *Streptantibioticus*, *Aeromicrobium*, *Marmoricola*, and *Arthrobacter* respectively.

To compare the variations in the microbial functional traits among the soil samples, a principal coordinate analysis (PCoA) (Supplementary fig. S1) was performed. The resulting PCoA plot showed that there was a relatively fair separation in functional traits between the soil samples which accounted for 75.8% of the total variance in the functional traits.

The microbial functional gene profiles were subjected to Principal Component Analysis (PCA) to determine the principal axes of variation among the rhizosphere and bulk soil samples. The two principal components axes captured (79.14%) and (20.86%) of the total variance respectively. The PCA scores plot (Supplementary Fig. S2) provided partial separation of the functional genes between rhizosphere soil *A.ampeloprasum* and uncultivated bulk soil samples which are driven along PCA1, indicating the difference in functional gene composition with regard to the effect of fertilization on the soil samples. Many of the microorganisms at the genus level reported in this work have earlier been proven to influence the functional genes in the soil. Among them are *Bradyrhizobium* [68]*, Pseudomonas* [31]*, Streptomyces* [108]*, Devosia* [107]*, Arthrobacter* [43]*, Microbacterium* [3]*, Sorangium* [24]*,* and *Cystobacter* [3].

As this investigation has shown, more microbial genera and families of microorganisms occupied the rhizosphere soils from the biofertilizer plot (G2) than the rhizosphere soil from the chemical fertilizer plot (G1) and bulk soils (G3) (Figs. 4 & 5). However, the microbial community’s low abundance in soils from G1 and G3 complies with the outcome of earlier findings from [41] who observed that bacterial diversity in soils treated with chemical fertilizers and bulk soil is lower than in that receiving organic fertilizers in barley-planted soil.

Nonetheless, there were no significant variations in the microbial alpha diversity estimation as depicted by the Shannon, Chao1, with evenness indices at the genus level (*P* > 0.05) (Table 2). The rhizosphere microbial community in *Allium ampeloprasum* cultivated with biofertilizer (G2) exhibited higher diversity and more even distribution compared to both the chemical fertilizer-treated (G1) and bulk soil samples (G3) (Fig. 5, Table 2, and supplementary Table 5).

As demonstrated by the PCoA graph (58.34% and 29.38%), the community of bacteria in the biofertilizer plot (G2) show a fair separation or little clustering compared to those of the chemical fertilizer plot (G1) and bulk soil samples (G3). This may imply that it is made up of microbial composition that is fairly different from the other two plots (Fig. 6). There is no significant difference (p-value > 0.05) in the microbial community taxonomic composition across the sampling plots as illustrated by the Euclidean dissimilarity matrix. The close proximities and clustering between group (G1 and G2) and (G1 and G3) supports the idea that rhizosphere’s microbiomes from chemical fertilizer and biofertilizer plots may share similarities with each other and with those in the bulk soils [55, 112, 114].

Total nitrogen and carbon significantly contributed 90.36% and 9.64% of the variability found in the microbial diversity, respectively, according to the redundancy analysis (RDA) results (Fig. 7, Table 3). All of the soil factors taken into consideration were essential in shaping the microorganism communities. If the magnitude of the vector of other environmental factors in our RDA plot is taken into consideration, it demonstrates that not only total nitrogen and carbon could influence the microbial communities.

Several soil factors were in correlation with compositional alterations in the structure of microorganisms’ community. The vector length of magnesium (Mg) as well as pH revealed a positive correlation with *Arthrobacter, Arthrobacter, Terrabacter, Rubrobacter, Sphingomonas* and *Pseudomonas* and the vector lengths of nitrate (N-NO_3_), carbon (C), ammonium (N-NH_4_) and total nitrate (Total-N) were positively correlated with *Roseimicrobium, Pseudoxanthomonas, Streptomyces, Variovorax, Devosia, Verrucomicrobium, Blastococcus, Polyangium, Dyadobacter, Sorangium, Minicystis, Haliangium and Cystobacter* while *Solirubrobacter, Microvirga, Nocardioides,* and *Methyloceanibacter* were positively correlated with the length of the clay vector (Fig. 7).

Biofertilizers have received increasing global attention over the past two decades, primarily due to their substantial benefits for plant development, environmental sustainability, and soil health, as they rely minimally or not at all on chemical inputs [49, 69]. Notably, the use of biofertilizer in this study has demonstrated distinct advantages over chemical fertilizers, including enhanced soil microbial diversity and improved nutrient cycling, which have collectively contributed to sustainable soil fertility [41, 44]. Similarly, biofertilizer reduce environmental pollution and input cost while promoting healthier plant growth, highlighting their roles as an eco-friendly alternative to chemical fertilizer [12, 13].

However, the mechanisms underlying the response of biofertilizers in the rhizosphere is keenly tied to the activities of incorporated beneficial microbes such as bacteria, fungi, and algae in interacting symbiotically with plant roots or the rhizosphere to enhance nutrient availability, mineralization, and uptake. These microorganisms are involved in different indispensable functions ranging from the conversion of atmospheric nitrogen into usable forms (nitrogen fixation), solubilization of insoluble phosphorus into soluble forms (phosphorus solubilization), and liberation of potassium from soil minerals, to the formation of mycorrhizal associations that expand surface area of roots for improved nutrient and water absorption [127]. Additionally, they produce growth-promoting substances like phytohormones (e.g., indole acetic acid), siderophores, and enzymes that facilitate nutrient cycling, improve soil structure, protect plants from pathogens, and boost overall plant growth and resilience.

Moreover, these rhizobacteria were able to suppress the competing soil microbes and successfully colonize the rhizosphere by producing antimicrobial compounds such as antibiotics, volatile organic compounds, lytic enzymes, as well as forming biofilms, outcompeting others for nutrients and space, and modulating the plant immune system [119, 121, 124].

It was discovered that the rhizosphere soils from chemical fertilizer-treated plot (G1) have a record of inorganic nitrogen fertilizer application as revealed by the soil history which could have led to the lowest microbial diversity in it. Our result complies with Dasgupta and Brahmaprakash [30] who concluded that the community of microorganisms in soil is influenced and affected by abiotic factors and chemical fertilizer which in turn alter the qualities of the soil. Nitrogen fertilizer inputs from the chemical fertilizers cause changes in the way microbial communities are assembled [20, 103]. Chemical fertilizers have been shown to lower soil pH, suppress microbial nutrients cycling, enzymatic activity, and organic matter as well as causing nutrients imbalance and toxicity which in turn restrict specific groups of microorganisms [32]. Furthermore, as stated by Pan et al. [94], the impacts of chemical fertilizers on soil microbial diversity lies in their capacity to supply readily available nutrients that can initially stimulate the growth of microorganisms, but their long-term or excessive use alters soil chemical properties, especially by lowering the soil pH through acidification, which adversely impacts microbial diversity and the balance of microbial communities, hence for an alternative such as biofertilizer is needed.

Biofertilizers influence the distribution of microbial functional genes in the soil by inducing beneficial microbes that carry genes which mediate essential nutrient cycling processes such as nitrogen fixation, phosphorus solubilization and potassium mobilization, thus increasing the abundance and activity of such functional genes in the rhizosphere. They stimulate the activities of microorganisms, enhance the levels of nutrient (N, P, K), generate plant growth-enhancing compounds (e.g., phytohormones, and siderophores), as well as enhancing the fertility of the soil. In contrast, chemical fertilizers offer easily accessed nutrients but these may diminish the activity of soil microbes and the abundance of functional genes in the soil, with time, as a result of the adverse effects on soil microbiota and biodiversity. Biofertilizers have been shown to positively impact the functional genes in the native microbial community by colonizing the rhizosphere and promoting microbial growth and by expression of vital genes which are involved in nutrient cycling, stress tolerance, nutrient acquisition and plant health promotion. Moreover, such gene-level improvement is accomplished by biofilm formation, competition and survival strategies, root colonization, and activation of microbial networks. These are vital in sustaining and enhancing the functional abundance, as well as the variety, to stabilize soil ecosystems and promote plant growth.

Overall, the highest abundance of microbial communities was observed in soil samples from the biofertilizer-treated plots (G2), compared to those from the chemical fertiliser and bulk soil samples (G2 and G3) respectively. Variations in the microbial abundance across the taxonomic levels (phylum, family and genus) between the samples, particularly within the biofertilizer-treated plot (G2), suggest that the rhizosphere could be a promising alternative way of isolating microorganisms for improving the productivity of plants, disease prevention, and soil biological environment enhancement [5, 33, 91] and biofertilizers could be a promising way of replenishing soil nutrients [9].

However, this result has also established that organic (biofertilizer) or inorganic fertilizers (chemical fertilizer) application could affect the accumulation of beneficial microbiomes and their functional traits around the rhizosphere of crops. Biofertilizers therefore reform and enrich the microbial functional gene pool more sustainably than chemical fertilizers, enhance the biological activities of soil, enhance the productivity of crops and reduces the negative impacts of biofertilizers on the environment.

Our results, confirmed our hypothesis that the rhizosphere of *A. ampeloprasum* under biofertilizer application boosted the recruitments of plant growth-promoting microbiome of agricultural importance.

Therefore, through the use of biofertilizers as an alternative soil fertilization system to chemical fertilizers, the restoration and maintenance of microbial diversity as well as their balance can support plant health, resilience to stress, and sustainable agricultural production.

However, this investigation showed many unclassified and unidentified bacterial groups (candidate division NC10), which may indicate the existence of possibly novel species because they cannot be categorized into the existing species [109]. It may be possible to identify new microorganisms (fungi and bacteria) that are of agricultural and biotechnological importance by mapping out methods to cultivate these microbes.

## Conclusion

This appears to be the first study, based on our knowledge, to adopt metagenomic analysis to examine how the introduction of chemical fertilizer and biofertilizer affects the organization of the soil community of microorganisms and the variety of microbial life from the rhizosphere of leek (*A. ampeloprasum*). Bacteria, fungi, viruses, and archaea make up the soil microbiome, with bacteria making up more than 85% of the entire microbial community.

Using the shotgun sequencing method, this investigation characterized beneficial rhizosphere microbiomes and their functional traits. This study has established that under biofertilizer application, the rhizosphere soils of crops host and serve as a homes for more agriculturally important microorganisms than under chemical fertilizer application.

In addition, biofertilizer boosted the recruitment of an important group of fungi ‘Ascomycota’. The diversity, richness and composition of the bacterial community accounted for the highest percentage of the microbial population among all samples of the soil, as reported in this research.

This study highlights the practical benefits of biofertilizer application over conventional chemical fertilizers in agricultural production. Even though chemical fertilizers supply readily available nutrients and short-term yield, they can suppress the growth as well as activities of indigenous microbial communities and, may degrade long-term soil fertility and environmental health.

In addition, the presence of diverse functional genes across the soil samples highlights the integral function of microbial communities in enhancing stress tolerance, plant defense nutrients cycling and overall growth of plants.

Therefore, the use of biofertilizer was shown to significantly enrich the soil with live and beneficial microbes that facilitate the nutrients cycling, decomposition of organic matter, improvement of soil structure, enhancement of beneficial plant–microbe interactions, nutrients solubilization and mineralization. Collectively, these activities will not only contribute to improved soil health but also create a more favorable environments for a diverse range of native beneficial rhizosphere or soil microbes to survive, reduced production input costs, and promote sustainable crop productivity.

The use of biofertilizer as a sustainable alternative to chemical fertilizer, offers a viable pathway toward environmentally responsible agricultures and long-term food security. The integration of microbial community composition with functional gene profiling underscores the valuable insights of rhizosphere microbiomes in soil health management and sustainable agricultural practices.

## Supplementary Information

Additional file 1

## Data Availability

The pure quality sequences are accessible on the NCBI-SRA dataset through the accession numbers: SRP537120 SRP537121 SRP537188.

## References

[1] Aasfar A, Meftah Kadmiri I, Azaroual SE, Lemriss S, Mernissi NE, Bargaz A, Zeroual Y, Hilali A. Agronomic advantage of bacterial biological nitrogen fixation on wheat plant growth under contrasting nitrogen and phosphorus regimes. Front Plant Sci. 2024;15:1388775. 10.3389/fpls.2024.1388775. 38779073 10.3389/fpls.2024.1388775 PMC11109382

[2] Acuña JJ, Rilling JI, Inostroza NG, Zhang Q, Wick LY, Sessitsch A, Jorquera MA. *Variovorax* sp. strain P1R9 applied individually or as part of bacterial consortia enhances wheat germination under salt stress conditions. Sci Rep. 2024;14(1):2070. 38267517 10.1038/s41598-024-52535-0 PMC10808091

[3] Adedayo AA, Fadiji AE, Babalola OO. Unraveling the functional genes present in rhizosphere microbiomes of *Solanum lycopersicum*. PeerJ. 2023;11: e15432. 37283894 10.7717/peerj.15432 PMC10241170

[4] Adeleke BS, Ayangbenro AS, Babalola OO. Genomic analysis of endophytic *Bacillus cereus* T4S and its plant growth-promoting traits. Plants. 2021;10(9):1776. 34579311 10.3390/plants10091776 PMC8467928

[5] Adeleke BS, Babalola OO. Roles of plant endosphere microbes in agriculture-a review. J Plant Growth Regul. 2022;41(4):1411–28.

[6] Affortit P, Ahmed MA, Grondin A, Delzon S, Carminati A, Laplaze L. Keep in touch: the soil–root hydraulic continuum and its role in drought resistance in crops. J Exp Bot. 2024;75(2):584–93. 37549338 10.1093/jxb/erad312

[7] Ahamad L, Shahid M, Danish M. Microbial biofertilizers: an environmentally-friendly approach to sustainable agriculture. In: Dar GH, Bhat RA, Mehmood MA, editors. Microbiomes for the management of agricultural sustainability. Springer: Switzerland; 2023. pp. 167–182. 10.1007/978-3-031-32967-8_9.

[8] Ajilogba CF, Babalola OO, Adebola P, Adeleke R. Bambara groundnut rhizobacteria antimicrobial and biofertilization potential. Front Plant Sci. 2022;13: 854937. 35909751 10.3389/fpls.2022.854937 PMC9326403

[9] Akanmu AO, Olowe OM, Phiri AT, Nirere D, Odebode AJ, Karemera Umuhoza NJ, Asemoloye MD, Babalola OO. Bioresources in organic farming: implications for sustainable agricultural systems. Horticulturae. 2023;9(6):659.

[10] Akinola SA, Ayangbenro AS, Babalola OO. The diverse functional genes of maize rhizosphere microbiota assessed using shotgun metagenomics. J Sci Food Agric. 2021;101(8):3193–201. 33215702 10.1002/jsfa.10948

[11] Alam A, Jahan AAA, Bari MS, Khandokar L, Mahmud MH, Junaid M, Chowdhury MS, Khan MF, Seidel V, Haque MA. Allium vegetables: traditional uses, phytoconstituents, and beneficial effects in inflammation and cancer. Crit Rev Food Sci Nutr. 2023;63(23):6580–614. 10.1080/10408398.2022.2036094. 35170391 10.1080/10408398.2022.2036094

[12] Alnaass NS, Agil HK, Alyaseer NA, Abubaira M, Ibrahim HK. The effect of biofertilization on plant growth and its role in reducing soil pollution problems with chemical fertilizers. Afr J Adv Pure Appl Sci (AJAPAS). 2023;2(3):387–400.

[13] Areej A, Usama M, Zulfiqar U, Sarwar F, Ashiq A. Sustainable agriculture development: the role of biofertilizers in soil fertility and crop yield improvement. Appl Agric Sci. 2024;2(1):1–5.

[14] Babalola OO, Akanmu AO, Fadiji AE. Dataset of shotgun metagenomic evaluation of lettuce (*Lactuta sativa* L.) rhizosphere microbiome. Data Brief. 2023;48: 109214. 10.1016/j.dib.2023.109214. 37228418 10.1016/j.dib.2023.109214 PMC10205420

[15] Babalola OO, Enagbonma BJ. Dataset of shotgun metagenomic evaluation of *Sorghum bicolor* rhizosphere microbiome in soils preceded by *Glycine max*. Data Brief. 2025;58: 111270. 39906131 10.1016/j.dib.2025.111270 PMC11791250

[16] Babalola OO, Olowe OM, Ayangbenro AS. Shotgun metagenomics dataset of *Striga hermonthica*-infested maize (*Zea mays* L.) rhizospheric soil microbiome. Data Brief. 2023;48: 109132. 10.1016/j.dib.2023.109132. 37383793 10.1016/j.dib.2023.109132 PMC10293916

[17] Baquy M, Li J-Y, Nkoh JN, Biswash MR, Xu R-K. Determining critical soil pH for phosphorus uptake efficiency in an acidic Ultisol for maize. Egypt J Soil Sci. 2024;64(4):1525–36.

[18] Breiman L. Random forests. Mach Learn. 2001;45(1):5–32.

[19] Buchfink B, Xie C, Huson DH. Fast and sensitive protein alignment using DIAMOND. Nat Methods. 2015;12(1):59–60. 25402007 10.1038/nmeth.3176

[20] Carrascosa A, Pascual JA, López-García Á, Romo-Vaquero M, De Santiago A, Ros M, Petropoulos SA, Alguacil MDM. Effects of inorganic and compost tea fertilizers application on the taxonomic and functional microbial diversity of the purslane rhizosphere. Front Plant Sci. 2023;14:1159823. 37152179 10.3389/fpls.2023.1159823 PMC10159062

[21] Chaudhary P, Singh S, Chaudhary A, Sharma A, Kumar G. Overview of biofertilizers in crop production and stress management for sustainable agriculture. Front Plant Sci. 2022;13: 930340. 36082294 10.3389/fpls.2022.930340 PMC9445558

[22] Chen L-J, Wu X-Q, Xu Y, Wang B-L, Liu S, Niu J-F, Wang Z. Microbial diversity and community structure changes in the rhizosphere soils of *Atractylodes lancea* from different planting years. Plant Signal Behav. 2021;16(2):1854507. 33289592 10.1080/15592324.2020.1854507 PMC7849755

[23] Chen S. Ultrafast one-pass FASTQ data preprocessing, quality control, and deduplication using fastp. Imeta. 2023;2(2): e107. 38868435 10.1002/imt2.107 PMC10989850

[24] Chen Y-P, Huang H-Y, Tsai C-F, Young C-C. Impact of fertilization and seasonal changes on paddy soil: unveiling the interplay between agricultural practices, enzyme activity, and gene diversity. Agriculture (Basel). 2024;14(8):1424.

[25] Chen Y, Xu T, Fu W, Hu Y, Hu H, You L, Chen B. Soil organic carbon and total nitrogen predict large-scale distribution of soil fungal communities in temperate and alpine shrub ecosystems. Eur J Soil Biol. 2021;102: 103270.

[26] Commichaux S, Shah N, Ghurye J, Stoppel A, Goodheart JA, Luque GG, Cummings MP, Pop M. A critical assessment of gene catalogs for metagenomic analysis. Bioinformatics. 2021;37(18):2848–57. 33792639 10.1093/bioinformatics/btab216 PMC8479683

[27] Cui H, Ou Y, Wang L, Yan B, Li Y, Bao M. Dissolved organic carbon, a critical factor to increase the bioavailability of phosphorus during biochar-amended aerobic composting. J Environ Sci (China). 2022;113:356–64. 34963543 10.1016/j.jes.2021.06.019

[28] Darjee S, Alekhya G, Sudarshan S, Padhan SR, Rajareddy G, Baishya M, Dash AK. Enhancing crop productivity with biofertilizers: exploring multifunctional benefits. Microbiol Res J Int. 2024;34(9):101–12.

[29] Das R, Bharadwaj P, Thakur D. Insights into the functional role of Actinomycetia in promoting plant growth and biocontrol in tea (*Camellia sinensis*) plants. Arch Microbiol. 2024;206(2):65. 38227026 10.1007/s00203-023-03789-1

[30] Dasgupta D, Brahmaprakash G. Soil microbes are shaped by soil physico-chemical properties: a brief review of existing literature. Int J Plant Soil Sci. 2021;33(1):59–71. 10.9734/ijpss/2021/v33i130409.

[31] Dignam BE, O’Callaghan M, Condron LM, Kowalchuk GA, Van Nostrand JD, Zhou J, Wakelin SA. Effect of land use and soil organic matter quality on the structure and function of microbial communities in pastoral soils: implications for disease suppression. PLoS ONE. 2018;13(5): e0196581. 29734390 10.1371/journal.pone.0196581 PMC5937765

[32] Dincă LC, Grenni P, Onet C, Onet A. Fertilization and soil microbial community: a review. Appl Sci. 2022;12(3):1198.

[33] Dlamini SP, Akanmu AO, Babalola OO. Rhizospheric microorganisms: the gateway to a sustainable plant health. Front Sustain Food Syst. 2022;6: 925802.

[34] Dlamini SP, Akanmu AO, Fadiji AE, Babalola OO. Maize rhizosphere modulates the microbiome diversity and community structure to enhance plant health. Saudi J Biol Sci. 2023;30(1): 103499. 36419926 10.1016/j.sjbs.2022.103499 PMC9677207

[35] Du S, Zhao X, Zhang Y, Shu C, Shen J-P. Different responses of soil fauna gut and plant rhizosphere microbiomes to manure applications. Soil Ecol Lett. 2024;6(2): 230196.

[36] Du T-Y, He H-Y, Zhang Q, Lu L, Mao W-J, Zhai M-Z. Positive effects of organic fertilizers and biofertilizers on soil microbial community composition and walnut yield. Appl Soil Ecol. 2022;175: 104457.

[37] Du T, Hu Q, He H, Mao W, Yang Z, Chen H, Sun L, Zhai M. Long-term organic fertilizer and biofertilizer application strengthens the associations between soil quality index, network complexity, and walnut yield. Eur J Soil Biol. 2023;116: 103492.

[38] Ekenwosu J, Okorie P, Nzenwa P. Stress and struggles of soil biodiversity in the global innovative technology for food sustainability. Trop Environ Biol Technol. 2024;2(2):72–9.

[39] Enagbonma BJ, Ajilogba CF, Babalola OO. Metagenomic profiling of bacterial diversity and community structure in termite mounds and surrounding soils. Arch Microbiol. 2020;202(10):2697–709. 32725600 10.1007/s00203-020-01994-w

[40] Enagbonma BJ, Babalola OO. Unveiling plant-beneficial function as seen in bacteria genes from termite mound soil. J Soil Sci Plant Nutr. 2020;20(2):421–30.

[41] Enagbonma BJ, Fadiji AE, Babalola OO. Anthropogenic fertilization influences a shift in barley rhizosphere microbial communities. PeerJ. 2024;12: e17303. 10.7717/peerj.17303. 39006020 10.7717/peerj.17303 PMC11246026

[42] Enebe MC, Babalola OO. Effects of inorganic and organic treatments on the microbial community of maize rhizosphere by a shotgun metagenomics approach. Ann Microbiol. 2020;70:1–10.

[43] Enebe MC, Babalola OO. Functional diversity of bacterial communities in the rhizosphere of maize grown on a soil under organic and inorganic fertilization. Sci Afr. 2022;16: e01212.

[44] Fadiji AE, Ayangbenro AS, Babalola OO. Metagenomic profiling of the community structure, diversity, and nutrient pathways of bacterial endophytes in maize plant. Antonie Van Leeuwenhoek. 2020;113(11):1559–71. 32803452 10.1007/s10482-020-01463-w

[45] Fadiji AE, Ayangbenro AS, Babalola OO. Unveiling the putative functional genes present in root-associated endophytic microbiome from maize plant using the shotgun approach. J Appl Genet. 2021;62(2):339–51. 10.1007/s13353-021-00611-w. 33486715 10.1007/s13353-021-00611-w

[46] Fadiji AE, Kanu JO, Babalola OO. Metagenomic profiling of rhizosphere microbial community structure and diversity associated with maize plant as affected by cropping systems. Int Microbiol. 2021;24(3):325–35. 33666787 10.1007/s10123-021-00169-x

[47] Fan W, Tang F, Wang J, Dong J, Xing J, Shi F. Drought-induced recruitment of specific root-associated bacteria enhances adaptation of alfalfa to drought stress. Front Microbiol. 2023;14:1114400. 36910228 10.3389/fmicb.2023.1114400 PMC9995459

[48] Feng Q, Liang S, Jia H, Stadlmayr A, Tang L, Lan Z, Zhang D, Xia H, Xu X, Jie Z. Gut microbiome development along the colorectal adenoma–carcinoma sequence. Nat Commun. 2015;6(1):6528. 25758642 10.1038/ncomms7528

[49] Fitriatin BN, Amanda AP, Kamaluddin NN, Khumairah FH, Sofyan ET, Yuniarti A, Turmuktini T. Some soil biological and chemical properties as affected by biofertilizers and organic ameliorants application on paddy rice. Eurasian J Soil Sci (EJSS). 2021;10(2):105–10.

[50] Freire MHC, Sousa GG, Viana TVA, Lessa CIN, Costa FHR. Soil chemical attributes under combinations of organic fertilizing and water salinity. Trop Agric Res. 2023;53:1. 10.1590/1983-40632023v5375156.

[51] Fu G, He Y. Responses of soil fungal and bacterial communities to long-term organic and inorganic nitrogenous fertilizers in an alpine agriculture. Appl Soil Ecol. 2024;201: 105498. 10.1016/j.apsoil.2024.105498.

[52] Garmay AV, Oskolok KV, Monogarova OV, Demidov MI. Determination of ammonium and nitrate in soils by digital colorimetry. Environ Monit Assess. 2024;196(10): 948. 10.1007/s10661-024-13068-1. 39292405 10.1007/s10661-024-13068-1

[53] Guo K, Yang J, Yu N, Luo L, Wang E. Biological nitrogen fixation in cereal crops: progress, strategies, and perspectives. Plant Commun. 2023. 10.1016/j.xplc.2022.100499. 36447432 10.1016/j.xplc.2022.100499 PMC10030364

[54] Hassoun A, Al-Muhannadi K, Hassan HF, Hamad A, Khwaldia K, Buheji M, Al Jawaldeh A. From acute food insecurity to famine: how the 2023/2024 war on Gaza has dramatically set back sustainable development goal 2 to end hunger. Front Sustain Food Syst. 2024;8: 1402150.

[55] Herre M, Heinze S, Heitkötter J, Marschner B. Different factors control organic matter degradation in bulk and rhizosphere soil from the top-and subsoils of three forest stands. Soil Biol Biochem. 2022;172: 108775.

[56] Huang Y. Illumina-based analysis of endophytic bacterial diversity of four *Allium* species. Sci Rep. 2019;9(1): 15271. 31649302 10.1038/s41598-019-51707-7 PMC6813343

[57] Huson DH, Auch AF, Qi J, Schuster SC. Megan analysis of metagenomic data. Genome Res. 2007;17(3):377–86. 17255551 10.1101/gr.5969107 PMC1800929

[58] Jana B, Chattopadhyay R, Das R, Kanthal S. Bio-fertilizer: an alternative to chemical fertilizer in agriculture. Int J Res Agron. 2024;7(4):144–9.

[59] Ji Z, Liu Y-Y, Liu F-C, Yao Y, Zhu C-Y, Yi X-C, Chen B, Xiao G-L. Effects of drought stress on rhizosphere soil bacterial community of potato throughout the reproductive period. J South Agric. 2023;54:1953–65.

[60] Jiao N, Song X, Song R, Yin D, Deng X. Diversity and structure of the microbial community in rhizosphere soil of *Fritillaria ussuriensis* at different health levels. PeerJ. 2022;10: e12778. 35127284 10.7717/peerj.12778 PMC8796711

[61] Jin F, Xu L, Xu H, Yang Q, Feng B. *Streptomyces toxytricini*, a biocontrol plant growth-promoting bacterium against smut of broomcorn millet (*Panicum miliaceum* L.). Biol Control. 2025;204: 105743.

[62] Jote CA. The impacts of using inorganic chemical fertilizers on the environment and human health. Org Med Chem Int J. 2023;13: 555864.

[63] Kanehisa M, Furumichi M, Tanabe M, Sato Y, Morishima K. KEGG: new perspectives on genomes, pathways, diseases and drugs. Nucleic Acids Res. 2017;45(1):D353–61. 27899662 10.1093/nar/gkw1092 PMC5210567

[64] Kanehisa M, Goto S, Hattori M, Aoki-Kinoshita KF, Itoh M, Kawashima S, Katayama T, Araki M, Hirakawa M. From genomics to chemical genomics: new developments in KEGG. Nucleic Acids Res. 2006;34(suppl_1):D354–7. 10.1093/nar/gkj102. 16381885 10.1093/nar/gkj102 PMC1347464

[65] Karimi E, Aliasgharzad N, Esfandiari E, Hassanpouraghdam MB, Neu TR, Buscot F, Reitz T, Breitkreuz C, Tarkka MT. Biofilm forming rhizobacteria affect the physiological and biochemical responses of wheat to drought. AMB Express. 2022;12(1):93. 35834031 10.1186/s13568-022-01432-8 PMC9283637

[66] Karlsson FH, Fåk F, Nookaew I, Tremaroli V, Fagerberg B, Petranovic D, Bäckhed F, Nielsen J. Symptomatic atherosclerosis is associated with an altered gut metagenome. Nat Commun. 2012;3(1):1245. 23212374 10.1038/ncomms2266 PMC3538954

[67] Karlsson FH, Tremaroli V, Nookaew I, Bergström G, Behre CJ, Fagerberg B, Nielsen J, Bäckhed F. Gut metagenome in European women with normal, impaired and diabetic glucose control. Nature. 2013;498(7452):99–103. 23719380 10.1038/nature12198

[68] Klepa MS, diCenzo GC, Hungria M. Comparative genomic analysis of Bradyrhizobium strains with natural variability in the efficiency of nitrogen fixation, competitiveness, and adaptation to stressful edaphoclimatic conditions. Microbiol Spectr. 2024;12(7):e00260-e224. 38842312 10.1128/spectrum.00260-24 PMC11218460

[69] Kumar A, Saharan BS, Parshad J, Gera R, Choudhary J, Yadav R. Revolutionizing Indian agriculture: the imperative of advanced biofertilizer technologies for sustainability. Discov Agric. 2024;2(1):24.

[70] Kumar M, Mishra S, Dixit V, Kumar M, Agarwal L, Chauhan PS, Nautiyal CS. Synergistic effect of *Pseudomonas putida* and *Bacillus amyloliquefaciens* ameliorates drought stress in chickpea (*Cicer arietinum* L.). Plant Signal Behav. 2016;11(1): e1071004. 26362119 10.1080/15592324.2015.1071004 PMC4871671

[71] Kumar S, Sindhu SS, Kumar R. Biofertilizers: an ecofriendly technology for nutrient recycling and environmental sustainability. Curr Res Microb Sci. 2022;3: 100094. 35024641 10.1016/j.crmicr.2021.100094 PMC8724949

[72] Langmead B, Salzberg SL. Fast gapped-read alignment with Bowtie 2. Nat Methods. 2012;9(4):357–9. 22388286 10.1038/nmeth.1923 PMC3322381

[73] Lei Z, Zhang K, Li C, Jiao T, Wu J, Wei Y, Tian K, Li C, Tang D, Davis DI. Ruminal metagenomic analyses of goat data reveals potential functional microbiota by supplementation with essential oil-cobalt complexes. BMC Microbiol. 2019;19:1–10. 30717674 10.1186/s12866-019-1400-3 PMC6362596

[74] Lema NK, Gemeda MT, Woldesemayat AA. Recent advances in metagenomic approaches, applications, and challenges. Curr Microbiol. 2023;80(11):347. 37733134 10.1007/s00284-023-03451-5

[75] Li D, Luo R, Liu C-M, Leung C-M, Ting H-F, Sadakane K, Yamashita H, Lam T-W. MEGAHIT v1. 0: a fast and scalable metagenome assembler driven by advanced methodologies and community practices. Methods. 2016;102:3–11. 27012178 10.1016/j.ymeth.2016.02.020

[76] Li J, Jia H, Cai X, Zhong H, Feng Q, Sunagawa S, Arumugam M, Kultima JR, Prifti E, Nielsen T. An integrated catalog of reference genes in the human gut microbiome. Nat Biotechnol. 2014;32(8):834–41. 24997786 10.1038/nbt.2942

[77] Li L, Hu Z, Tan G, Fan J, Chen Y, Xiao Y, Wu S, Zhi Q, Liu T, Yin H. Enhancing plant growth in biofertilizer-amended soil through nitrogen-transforming microbial communities. Front Plant Sci. 2023;14:1259853. 38034579 10.3389/fpls.2023.1259853 PMC10683058

[78] Li M, Zhu Q, Liu H, Xia X, Huang D. Method for detecting soil total nitrogen contents based on pyrolysis and artificial olfaction. Int J Agric Biol Eng. 2022;15(3):167–76.

[79] Lombardino J, Bijlani S, Singh NK, Wood JM, Barker R, Gilroy S, Wang CC, Venkateswaran K. Genomic characterization of potential plant growth-promoting features of *Sphingomonas* strains isolated from the International Space Station. Microbiol Spectr. 2022;10(1):e01994-e1921. 35019675 10.1128/spectrum.01994-21 PMC8754149

[80] Lopes LD, Futrell SL, Bergmeyer E, Hao J, Schachtman DP. Root exudate concentrations of indole-3-acetic acid (IAA) and abscisic acid (ABA) affect maize rhizobacterial communities at specific developmental stages. FEMS Microbiol Ecol. 2023;99(3): fiad019. 10.1093/femsec/fiad019. 36861302 10.1093/femsec/fiad019

[81] Ma W, Tang S, Dengzeng Z, Zhang D, Zhang T, Ma X. Root exudates contribute to belowground ecosystem hotspots: a review. Front Microbiol. 2022;13: 937940. 36274740 10.3389/fmicb.2022.937940 PMC9581264

[82] Ma Y, Shen S, Wan C, Wang S, Yang F, Zhang K, Gao W. Organic fertilizer substitution over six years improves the productivity of garlic, bacterial diversity, and microbial communities network complexity. Appl Soil Ecol. 2023;182: 104718.

[83] Majewska M, Słomka A, Hanaka A. Siderophore-producing bacteria from Spitsbergen soils—novel agents assisted in bioremediation of the metal-polluted soils. Environ Sci Pollut Res. 2024;31(22):32371–81. 10.1007/s11356-024-33356-0 PMC11133149 38652189

[84] Mandal SK, Kambhampati JM, Sharma VS, Sirisha VSL, Sharvani P, Reddy CN, Yadavalli R, Mishra B. Gene prediction through metagenomics. In: Shah MP editor. Microbial Metagenomics in Effluent Treatment Plant. Elsevier; 2024. pp. 63–92. 10.1016/B978-0-443-13531-6.00013-6

[85] Meng C, Xing Y, Ding Y, Zhang Q, Di H, Tang C, Xu J, Li Y. Soil acidification induced variation of nitrifiers and denitrifiers modulates N2O emissions in paddy fields. Sci Total Environ. 2023;882: 163623. 10.1016/j.scitotenv.2023.163623. 37086999 10.1016/j.scitotenv.2023.163623

[86] Mozaffari H, Moosavi AA, Baghernejad M, Cornelis W. Revisiting soil texture analysis: introducing a rapid single-reading hydrometer approach. Measurement. 2024;228: 114330. 10.1016/j.measurement.2024.114330.

[87] Mozaffari H, Moosavi AA, Dematte JA. Estimating particle-size distribution from limited soil texture data: introducing two new methods. Biosyst Eng. 2022;216:198–217.

[88] Mukherjee A, Singh BN, Kaur S, Sharma M, de Araújo ASF, de Araujo Pereira AP, Morya R, Puopolo G, Melo VMM, Verma JP. Unearthing the power of microbes as plant microbiome for sustainable agriculture. Microbiol Res. 2024;286: 127780. 38970905 10.1016/j.micres.2024.127780

[89] Nsengimana V, de Dieu Nsenganeza J, Hagenimana T, Dekoninck W. Impact of chemical fertilizers on diversity and abundance of soil-litter arthropod communities in coffee and banana plantations in southern Rwanda. Curr Res Environ Sustain. 2023;5: 100215. 10.1016/j.crsust.2023.100215.

[90] Oksanen J, Kindt R, Legendre P, O’Hara B, Simpson G, Solymos P, Stevens M, Wagner H. vegan: an R package for community ecologists (R Package Version). World Agrofor (ICRAF). 2016;14(41):37.

[91] Olanrewaju OS, Babalola OO. The rhizosphere microbial complex in plant health: a review of interaction dynamics. J Integr Agric. 2022;21(8):2168–82.

[92] Omotayo OP, Igiehon ON, Babalola OO. Microbial genes of agricultural importance in maize rhizosphere unveiled through shotgun metagenomics. Span J Soil Sci. 2022;12: 10427.

[93] Ozma MA, Abbasi A, Ahangarzadeh Rezaee M, Hosseini H, Hosseinzadeh N, Sabahi S, Noori SMA, Sepordeh S, Khodadadi E, Lahouty M. A critical review on the nutritional and medicinal profiles of garlic’s (*Allium sativum* L.) bioactive compounds. Food Rev Int. 2023;39(9):6324–61.

[94] Pan X, Yu H-J, Zhang B, Guan Y-Q, Zhang N, Du H-L, Liu F-M, Yu J, Wang Q-J, Liu J. Effects of organic fertilizer replacement on the microbial community structure in the rhizosphere soil of soybeans in albic soil. Sci Rep. 2025;15(1): 12271. 40210963 10.1038/s41598-025-96463-z PMC11986170

[95] Penuelas J, Coello F, Sardans J. A better use of fertilizers is needed for global food security and environmental sustainability. Agric Food Secur. 2023;12(1):1–9.36883120

[96] Purwestri YA, Nuringtyas TR, Wibowo AT, Nugrahapraja H, Salsinha YCF, Sebastian A, Nurbaiti S, Kumalasari N, Annisa RR, Manik Putri SP. Application of osmoprotectant enhance tolerance to drought stress in rice and trigger changes in root microbial composition. J Plant Biochem Biotechnol. 2024. 10.1007/s13562-024-00933-w.

[97] Qian S, Xu Y, Zhang Y, Wang X, Niu X, Wang P. Effect of AMF inoculation on reducing excessive fertilizer use. Microorganisms. 2024;12(8):1550. 10.3390/microorganisms12081550. 39203391 10.3390/microorganisms12081550 PMC11356082

[98] Rammali S, Rahim A, El Aalaoui M, Bencharki B, Dari K, Habach A, Abdeslam L, Khattabi A. Antimicrobial potential of *Streptomyces coeruleofuscus* SCJ isolated from microbiologically unexplored garden soil in Northwest Morocco. Sci Rep. 2024;14(1):3359. 38336871 10.1038/s41598-024-53801-x PMC10858231

[99] Rao CR. The use and interpretation of principal component analysis in applied research. Indian J Stat. 1964;26(4):329–58.

[100] Rochlani A, Dalwani A, Shaikh N, Shaikh N, Sharma S, Saraf M. Plant growth promoting rhizobacteria as biofertilizers: application in agricultural sustainability. Acta Sci Microbiol. 2022;5(4):12–21.

[101] Segata N, Izard J, Waldron L, Gevers D, Miropolsky L, Garrett WS, Huttenhower C. Metagenomic biomarker discovery and explanation. Genome Biol. 2011;12(6):R60. 21702898 10.1186/gb-2011-12-6-r60 PMC3218848

[102] Sharma I, Kashyap S, Agarwala N. Biotic stress-induced changes in root exudation confer plant stress tolerance by altering rhizospheric microbial community. Front Plant Sci. 2023;14:1132824. 36968415 10.3389/fpls.2023.1132824 PMC10036841

[103] Shen M-C, Zhang Y-Z, Bo G-D, Yang B, Wang P, Ding Z-Y, Wang Z-B, Yang J-M, Zhang P, Yuan X-L. Microbial responses to the reduction of chemical fertilizers in the rhizosphere soil of flue-cured tobacco. Front Bioeng Biotechnol. 2022;9: 812316. 10.3389/fbioe.2021.812316. 35087808 10.3389/fbioe.2021.812316 PMC8787768

[104] Singh K, Chandra R, Purchase D. Unraveling the secrets of rhizobacteria signaling in rhizosphere. Rhizosphere. 2022;21: 100484. 10.1016/j.rhisph.2022.100484.

[105] Sun C, Wang R, Tang G, Cai S, Shi H, Liu F, Xie H, Zhu J, Xiong Q. Integrated 16S and metabolomics revealed the mechanism of drought resistance and nitrogen uptake in rice at the heading stage under different nitrogen levels. Front Plant Sci. 2023;14: 1120584. 37089655 10.3389/fpls.2023.1120584 PMC10114610

[106] Suriyagoda L, Dandeniya WS, Kadupitiya HK, Madushan R, Senarathne S, Chandrajith R. Evaluation of exchangeable potassium concentration in Sri Lankan rice paddy soils with ICP-MS using CaCl2 extraction. Commun Soil Sci Plant Anal. 2024;55(22):3547–61. 10.1080/00103624.2024.2402807.

[107] Talwar C, Nagar S, Kumar R, Scaria J, Lal R, Negi RK. Defining the environmental adaptations of genus *Devosia*: insights into its expansive short peptide transport system and positively selected genes. Sci Rep. 2020;10(1): 1151. 31980727 10.1038/s41598-020-58163-8 PMC6981132

[108] Tiwari P, Ansari WA, Kumar SC, Tiwari PK, Kumar M, Chakdar H, Srivastava AK, Saxena AK, Shantikumar L. Genetic diversity and functional potential of *Streptomyces* spp. isolated from Pachmarhi Biosphere Reserve, India. Curr Microbiol. 2024;81(11): 397. 39377919 10.1007/s00284-024-03927-y

[109] Vanni C, Schechter MS, Acinas SG, Barberán A, Buttigieg PL, Casamayor EO, Delmont TO, Duarte CM, Eren AM, Finn RD. Unifying the known and unknown microbial coding sequence space. Elife. 2022;11: e67667. 10.7554/eLife.67667. 35356891 10.7554/eLife.67667 PMC9132574

[110] Villar E, Farrant GK, Follows M, Garczarek L, Speich S, Audic S, Bittner L, Blanke B, Brum JR, Brunet C. Environmental characteristics of Agulhas rings affect interocean plankton transport. Science. 2015;348(6237):1261447. 10.1126/science.1261447. 25999514 10.1126/science.1261447

[111] Wakung’oli M, Amoo AE, Enagbonma BJ, Babalola OO. Termite societies promote the taxonomic and functional diversity of archaeal communities in mound soils. Biology. 2020;9(6): 136. 32630446 10.3390/biology9060136 PMC7345372

[112] Wang C, Pan G, Lu X, Qi W. Phosphorus solubilizing microorganisms: potential promoters of agricultural and environmental engineering. Front Bioeng Biotechnol. 2023;11:1181078. 37251561 10.3389/fbioe.2023.1181078 PMC10213388

[113] Wang J, Liu L, Gao X, Hao J, Wang M. Elucidating the effect of biofertilizers on bacterial diversity in maize rhizosphere soil. PLoS ONE. 2021;16(4): e0249834. 33891590 10.1371/journal.pone.0249834 PMC8064744

[114] Wang Y, Dong L, Zhang M, Cui Y, Bai X, Song B, Zhang J, Yu X. Dynamic microbial community composition, co-occurrence pattern and assembly in rhizosphere and bulk soils along a coniferous plantation chronosequence. CATENA. 2023;223: 106914.

[115] Wu Q, Chen Y, Dou X, Liao D, Li K, An C, Li G, Dong Z. Microbial fertilizers improve soil quality and crop yield in coastal saline soils by regulating soil bacterial and fungal community structure. Sci Total Environ. 2024;949: 175127. 39084360 10.1016/j.scitotenv.2024.175127

[116] Xie T, Wu Q, Lu H, Hu Z, Luo Y, Chu Z, Luo F. Functional perspective of leeks: active components, health benefits and action mechanisms. Foods. 2023;12(17): 3225. 37685158 10.3390/foods12173225 PMC10486880

[117] Yan H, Wu Y, He G, Wen S, Yang L, Ji L. Fertilization regime changes rhizosphere microbial community assembly and interaction in *Phoebe bournei* plantations. Appl Microbiol Biotechnol. 2024;108(1):417. 38995388 10.1007/s00253-024-13106-5 PMC11245453

[118] Yang J, Liu X, Rong X, Jiang P, Xia Y, Xie G, Luo G, Yan X. Bio-organic fertilizer application improves cucumber growth, disease resistance, and soil fertility by regulating rhizosphere microbiomes. Plant Soil. 2025. 10.1007/s11104-025-07460-0.

[119] Yang L, Qian X, Zhao Z, Wang Y, Ding G, Xing X. Mechanisms of rhizosphere plant-microbe interactions: molecular insights into microbial colonization. Front Plant Sci. 2024;15:1491495. 39606666 10.3389/fpls.2024.1491495 PMC11600982

[120] Yang R, Sun Z, Gong Y, Zhou P, Zhang X, Wang J, Dong Q, Gao F. The impact of nutrient deficiency on the structure of soil microbial communities within a double-cropping system. Front Plant Sci. 2025;16:1487687. 39944178 10.3389/fpls.2025.1487687 PMC11814463

[121] Yang Z, Liu T, Fan J, Chen Y, Wu S, Li J, Liu Z, Yang Z, Li L, Liu S. Biocontrol agents modulate phyllosphere microbiota interactions against pathogen *Pseudomonas syringae*. Environ Sci Ecotechnol. 2024;21: 100431. 38883559 10.1016/j.ese.2024.100431 PMC11177076

[122] Zádrapová D, Chakraborty A, Žáček P, Korecký J, Bhar A, Roy A. Exploring the rhizospheric microbial communities under long-term precipitation regime in Norway spruce seed orchard. Int J Mol Sci. 2024;25(17):9658. 39273604 10.3390/ijms25179658 PMC11395193

[123] Zang Z, Li Y, Wang Y, Zhang Y, Deng S, Guo X, Yang K, Zhao W. Contrasting roles of plant, bacterial, and fungal diversity in soil organic carbon accrual during ecosystem restoration: a meta-analysis. Sci Total Environ. 2024;930: 172767. 38670358 10.1016/j.scitotenv.2024.172767

[124] Zboralski A, Filion M. Genetic factors involved in rhizosphere colonization by phytobeneficial *Pseudomonas* spp. Comput Struct Biotechnol J. 2020;18:3539–54. 33304453 10.1016/j.csbj.2020.11.025 PMC7711191

[125] Zeller G, Tap J, Voigt AY, Sunagawa S, Kultima JR, Costea PI, Amiot A, Böhm J, Brunetti F, Habermann N. Potential of fecal microbiota for early-stage detection of colorectal cancer. Mol Syst Biol. 2014;10(11): 766. 10.15252/msb.20145645. 25432777 10.15252/msb.20145645 PMC4299606

[126] Zhang R, Li Y, Zhao X, Degen AA, Lian J, Liu X, Li Y, Duan Y. Fertilizers have a greater impact on the soil bacterial community than on the fungal community in a sandy farmland ecosystem, Inner Mongolia. Ecol Indic. 2022;140: 108972.

[127] Zhao G, Zhu X, Zheng G, Meng G, Dong Z, Baek JH, Jeon CO, Yao Y, Xuan YH, Zhang J. Development of biofertilizers for sustainable agriculture over four decades (1980–2022). Geogr Sustain. 2024;5(1):19–28.

[128] Zhao W, Chen Z, Yang X, Sheng L, Mao H, Zhu S. Metagenomics reveal arbuscular mycorrhizal fungi altering functional gene expression of rhizosphere microbial community to enhance *Iris tectorum*’s resistance to Cr stress. Sci Total Environ. 2023;895: 164970. 10.1016/j.scitotenv.2023.164970. 37343864 10.1016/j.scitotenv.2023.164970

[129] Zhour H, Bray F, Dandache I, Marti G, Flament S, Perez A, Lis M, Cabrera-Bosquet L, Perez T, Fizames C. Wild wheat rhizosphere-associated plant growth-promoting bacteria exudates: effect on root development in modern wheat and composition. Int J Mol Sci. 2022;23(23):15248. 36499572 10.3390/ijms232315248 PMC9740669

