# Supplementary material for: Deciphering the influence of fertilization systems on the Allium ampeloprasum rhizosphere microbial diversity and community structure through a shotgun metagenomics profiling approach
Source: Environ Microbiome. 2025 Oct 6;20:126. doi: 10.1186/s40793-025-00771-w (PMC12502447; doi:10.1186/s40793-025-00771-w)
Supplement: Supplementary file 1 — Additional file 1 [file 40793_2025_771_MOESM1_ESM.docx]

# Biofertilizer promotes the microbial diversity and community structure of rhizosphere microbiome of *Allium ampeloprasun* when examined under different fertilisation system

**Supplementary**

## **Table S1**: Soil physicochemical properties of rhizosphere soil samples of *Allium ampeloprasum* rhizosphere (under chemical fertiliser and biofertilizer) and Bulk soil

| Sample ID | L1 | L2 | L3 | L4 | L9 | L10 | L11 | L12 | LB1 | LB2 | LB3 | LB4 |
| --- | --- | --- | --- | --- | --- | --- | --- | --- | --- | --- | --- | --- |
| Ph | 7.5 | 7.32 | 7.35 | 7.16 | 7.17 | 7.15 | 7.24 | 7.28 | 7.22 | 7.21 | 7.33 | 7.31 |
| P | 200 | 215 | 190 | 230 | 205 | 225 | 238 | 253 | 223 | 245 | 215 | 205 |
| L | 7.6 | 5.6 | 7.2 | 7.8 | 10.5 | 7.5 | 10 | 7.1 | 9.1 | 10.6 | 7.7 | 8 |
| Ca | 77 | 76.2 | 73.4 | 73 | 73 | 75.1 | 72.4 | 76.1 | 72.6 | 71.5 | 75.2 | 75 |
| Mg | 15.4 | 18.1 | 19.2 | 19 | 16.5 | 17 | 17.5 | 16.6 | 18 | 17.9 | 17 | 17.1 |
| Na | 0 | 0 | 0.2 | 0.2 | 0 | 0.4 | 0 | 0.2 | 0.4 | 0 | 0 | 0 |
| N-NO3 | 4.04 | 2.49 | 2.69 | 7.44 | 9.03 | 7.16 | 11.99 | 7.26 | 6.85 | 7.09 | 4.32 | 4.06 |
| N-NH4 | 1.45 | 1.05 | 1.3 | 1.5 | 2.05 | 1.55 | 1.75 | 1.55 | 1.15 | 1.2 | 1.5 | 1.85 |
| Total N | 442 | 424 | 395 | 409 | 475 | 487 | 534 | 422 | 442 | 492 | 375 | 350 |
| Sand | 79 | 79 | 79 | 79 | 76 | 81 | 79 | 79 | 77 | 76 | 79 | 78 |
| silt | 11 | 9 | 6 | 7 | 8 | 8 | 9 | 8 | 7 | 8 | 7 | 9 |
| Clay | 10 | 12 | 15 | 14 | 16 | 11 | 12 | 13 | 16 | 16 | 14 | 13 |
| C | 0.55 | 0.48 | 0.55 | 0.53 | 0.59 | 0.66 | 0.68 | 0.5 | 0.52 | 0.63 | 0.5 | 0.43 |
| S - Value | 6.711 | 6.513 | 6.031 | 5.88 | 6.746 | 6.061 | 6.509 | 5.739 | 5.878 | 6.678 | 6.067 | 5.803 |
| Moisture content | 3.7 | 4.9 | 6.6 | 9.9 | 8.7 | 4.8 | 4.2 | 6.6 | 6.2 | 4.6 | 4.1 | 5.7 |

## Table S2: Analysis of sequenced data of the shotgun metagenome from the rhizosphere of the Allium ampeloprasum rhizosphere and uncultivated bulk soil

| SampleID | Total len(bp) | Scaftigs num | Average len(bp) | N50 len(bp) | N90 len(bp) | Max len(bp) |
| --- | --- | --- | --- | --- | --- | --- |
| L1 | 207 133 575 | 279 989 | 739.79 | 696 | 526 | 37 428 |
| L2 | 220 163 088 | 293 663 | 749.71 | 708 | 527 | 18 728 |
| L3 | 230 004 647 | 305 830 | 752.07 | 709 | 528 | 29 278 |
| L4 | 189 398 882 | 254 560 | 744.02 | 697 | 525 | 16 528 |
| L9 | 285 268 861 | 316 496 | 901.33 | 794 | 533 | 1 006 233 |
| L10 | 163 046 484 | 223 983 | 727.94 | 674 | 523 | 46 653 |
| L11 | 224 304 831 | 310 609 | 722.15 | 678 | 524 | 29 791 |
| L12 | 190 484 290 | 254 888 | 747.33 | 695 | 525 | 52 627 |
| LB1 | 205 215 937 | 279 183 | 735.06 | 699 | 527 | 9 711 |
| LB2 | 289 466 119 | 384 519 | 752.8 | 716 | 529 | 24 859 |
| LB3 | 148 202 624 | 207 600 | 713.89 | 672 | 523 | 10 154 |
| LB4 | 166 948 562 | 228 005 | 732.21 | 689 | 525 | 15 500 |

Three groups, G1, G2, and G3, each consisting of 4 replicates. G1 (Soil samples from chemical fertiliser plot) = L1, L2, L3 and L4; G2(Soil samples from biofertiliser plot) = (L9, L10, L11 and L12); and G3(Soil samples from uncultivated bulk soils) = (LB1, LB2, LB3 and LB4).

## Table S3: Percentage (%) Abundance of Microbial Communities at the Phylum Level

| Phylum | G1 | G2 | G3 |
| --- | --- | --- | --- |
| Actinomycetota | 42.67 | 42.32 | 43.95 |
| Pseudomonadota | 15.27 | 16.08 | 14.02 |
| Acidobacteriota | 10.67 | 7.57 | 10.07 |
| Myxococcota | 1.98 | 4.27 | 2 |
| Chloroflexota | 8.02 | 6.93 | 8.14 |
| Bacteroidota | 0.4 | 2.47 | 0.35 |
| Verrucomicrobiota | 0.31 | 1.95 | 0.29 |
| Nitrososphaerota | 1.92 | 1.96 | 2.48 |
| Gemmatimonadota | 2.88 | 2.29 | 2.75 |
| Planctomycetota | 1.4 | 0.95 | 0.98 |
| Candidatus Rokuibacteriota | 1.01 | 0.79 | 0.99 |
| Bacillota | 0.68 | 0.9 | 1.1 |
| Nitrospirota | 1.31 | 1.08 | 1.29 |
| Thermomicrobiota | 1.23 | 1.03 | 1.28 |
| Candidatus Eisenbacteria | 0.17 | 0.11 | 0.15 |
| Cyanobacteriota | 0.26 | 0.23 | 0.25 |
| Thermodesulfobacteriota | 0.1 | 0.09 | 0.1 |
| Euryarchaeota | 0.06 | 0.05 | 0.07 |
| Armatimonadota | 0.05 | 0.04 | 0.05 |
| Deinococcota | 0.05 | 0.04 | 0.05 |
| Candidatus Tectomicrobia | 0.03 | 0.02 | 0.03 |
| Uroviricota | 0.01 | 0.02 | 0.01 |
| candidate division NC10 | 0.02 | 0.02 | 0.02 |
| Spirochaetota | 0.02 | 0.03 | 0.02 |
| Thermoproteota | 0.01 | 0.02 | 0.02 |
| Candidatus Dormiibacterota | 0.01 | 0.01 | 0.02 |
| Calditrichota | 0.02 | 0.01 | 0.02 |
| Bdellovibrionota | 0.01 | 0.01 | 0.01 |
| Rhodothermota | 0.02 | 0.01 | 0.02 |
| Candidatus Aminicenantes | 0.02 | 0.01 | 0.01 |
| Ignavibacteriota | 0.01 | 0.01 | 0.01 |
| Ascomycota | 0.01 | 0.01 | 0.01 |
| Nitrospinota | 0.01 | 0.01 | 0.01 |
| Candidatus Latescibacterota | 0.01 | 0.01 | 0.01 |
| Fibrobacterota | 0 | 0 | 0 |

G1 (Soil samples from chemical fertiliser plot)

G2(Soil samples from biofertiliser plot)

G3(Soil samples from uncultivated bulk soils)

## Table S4: Percentage (%) Abundance of Microbial Communities at the Family Level

| Family | G1 | G2 | G3 |
| --- | --- | --- | --- |
| Nocardioidaceae | 12.97 | 13.81 | 14.58 |
| Spirosomataceae | 0.01 | 1.78 | 0.01 |
| Verrucomicrobiaceae | 0.05 | 1.62 | 0.03 |
| Solirubrobacteraceae | 2.92 | 2.75 | 3.18 |
| Streptomycetaceae | 0.88 | 2.26 | 0.67 |
| Rubrobacteraceae | 2.06 | 1.08 | 1.93 |
| Kofleriaceae | 0.02 | 0.93 | 0.02 |
| Polyangiaceae | 0.07 | 0.95 | 0.07 |
| Micrococcaceae | 2.36 | 1.72 | 1.47 |
| Intrasporangiaceae | 2.25 | 2.27 | 2.18 |
| Microbacteriaceae | 1.39 | 2.09 | 0.94 |
| Comamonadaceae | 0.29 | 0.97 | 0.21 |
| Xanthomonadaceae | 0.21 | 0.83 | 0.13 |
| Pseudomonadaceae | 0.73 | 0.23 | 0.06 |
| Gaiellaceae | 1.79 | 1.84 | 2.05 |
| Hyphomicrobiaceae | 1.24 | 1.39 | 1.55 |
| Sphingomonadaceae | 1.53 | 1.53 | 1.46 |
| Nitrososphaeraceae | 1.17 | 1.2 | 1.47 |
| Archangiaceae | 0.15 | 0.6 | 0.19 |
| Nitrospiraceae | 1.14 | 0.94 | 1.13 |
| Geodermatophilaceae | 0.93 | 1.05 | 0.96 |
| Nitrobacteraceae | 0.87 | 1.1 | 0.99 |
| Nocardiaceae | 0.38 | 0.11 | 0.09 |
| Paracoccaceae | 0.51 | 0.47 | 0.17 |
| Devosiaceae | 0.04 | 0.33 | 0.03 |
| Gemmataceae | 0.52 | 0.24 | 0.3 |
| Methylobacteriaceae | 0.6 | 0.62 | 0.67 |
| Thermoactinomycetaceae | 0.25 | 0.36 | 0.44 |
| Xanthobacteraceae | 0.44 | 0.51 | 0.5 |
| Pseudonocardiaceae | 0.58 | 0.58 | 0.62 |
| Promicromonosporaceae | 0.21 | 0.13 | 0.05 |
| Chitinophagaceae | 0.09 | 0.2 | 0.08 |
| Longimicrobiaceae | 0.22 | 0.14 | 0.14 |
| Bacillaceae | 0.22 | 0.32 | 0.38 |
| Conexibacteraceae | 0.32 | 0.27 | 0.33 |

G1 (Soil samples from chemical fertiliser plot)

G2(Soil samples from biofertiliser plot)

G3(Soil samples from uncultivated bulk soils)

## Table S5: Percentage (%) Abundance of Microbial Communities at the Genus Level

| Genus | G1 | G2 | G3 |
| --- | --- | --- | --- |
| *Nocardioides* | 11.39 | 11.94 | 12.72 |
| *Dyadobacter* | 0 | 1.68 | 0 |
| *Verrucomicrobium* | 0.01 | 1.12 | 0 |
| *Haliangium* | 0.01 | 0.9 | 0.01 |
| *Streptomyces* | 0.67 | 1.63 | 0.57 |
| *Solirubrobacter* | 2.41 | 2.28 | 2.59 |
| *Pseudomonas* | 0.72 | 0.22 | 0.05 |
| *Pseudoxanthomonas* | 0.07 | 0.59 | 0.03 |
| *Variovorax* | 0.08 | 0.58 | 0.05 |
| *Gaiella* | 1.4 | 1.47 | 1.59 |
| *Sphingomonas* | 1.45 | 1.41 | 1.39 |
| *Arthrobacter* | 1.25 | 0.86 | 0.8 |
| *Nitrospira* | 1.11 | 0.92 | 1.09 |
| *Cystobacter* | 0.06 | 0.39 | 0.08 |
| *Streptantibioticus* | 0.09 | 0.39 | 0.01 |
| *Microbacterium* | 0.56 | 0.95 | 0.36 |
| *Minicystis* | 0.01 | 0.32 | 0.01 |
| *Rhodococcus* | 0.35 | 0.08 | 0.05 |
| *Knoellia* | 0.78 | 0.82 | 0.7 |
| *Bradyrhizobium* | 0.66 | 0.85 | 0.77 |
| *Devosia* | 0.03 | 0.32 | 0.03 |
| *Rubrobacter* | 0.63 | 0.34 | 0.59 |
| *Sorangium* | 0.03 | 0.27 | 0.03 |
| *Paracoccus* | 0.39 | 0.24 | 0.12 |
| *Nitrosocosmicus* | 0.66 | 0.77 | 0.86 |
| *Marmoricola* | 0.56 | 0.71 | 0.81 |
| *Agromyces* | 0.72 | 0.88 | 0.5 |
| *Methyloceanibacter* | 0.51 | 0.61 | 0.69 |
| *Microvirga* | 0.55 | 0.56 | 0.62 |
| *Roseimicrobium* | 0.02 | 0.22 | 0 |
| *Blastococcus* | 0.51 | 0.57 | 0.5 |
| *Pseudolabrys* | 0.4 | 0.48 | 0.46 |
| *Aeromicrobium* | 0.37 | 0.44 | 0.3 |
| *Terrabacter* | 0.5 | 0.4 | 0.48 |
| *Polyangium* | 0.02 | 0.19 | 0.02 |

G1 (Soil samples from chemical fertiliser plot)

G2(Soil samples from biofertiliser plot)

G3(Soil samples from uncultivated bulk soils)

## **Table S6:** Raw relative abundance of disease incidence across the soil samples with ANOVA analysis

| SAMPLE  ID | Bacterial leaf blight | Blast | Black rot | Fusarium ear blight | Fire blight | Bacterial  wilt | Soft rot | Bacterial speck | Fusarium head blight | Canker |
| --- | --- | --- | --- | --- | --- | --- | --- | --- | --- | --- |
| G1 | 415.25± 9.64a | 361.00±18.17a | 375.50± 5.80a | 313.50±16.66a | 252.00±10.23a | 209.75± 9.67a | 203.00± 6.22a | 179.50±12.79a | 181.75± 9.07a | 105.25±4.86a |
| G2 | 418.25±20.61a | 349.75±53.43a | 364.00± 6.98a | 305.50±37.68a | 246.00±26.09a | 197.00±15.03a | 196.25±14.01a | 168.75±18.63a | 176.50±25.67a | 101.75±3.95a |
| G3 | 410.75± 2.87a | 357.25±10.14a | 368.00±10.36a | 313.50± 8.96a | 249.00± 8.76a | 201.00± 4.32a | 204.50± 4.36a | 174.00± 5.48a | 181.75± 6.18a | 106.25±4.92a |

G1 (Soil samples from chemical fertiliser plot)

G2(Soil samples from biofertiliser plot)

G3(Soil samples from uncultivated bulk soil).

## Table S7: Raw relative abundance of functional genes across the soil samples with ANOVA analysis

| Group | KO_ID | G1 | | G2 | G3 |
| --- | --- | --- | --- | --- | --- |
| aqpZ | K06188 | 1079.80±49.897a | | 1042.42±39.435a | 1036.34±42.834a |
| betA | K00108 | 1233.78±66.689a | | 1221.59±161.149a | 1232.24±45.653a |
| betB | K00130 | 3293.18±245.266a | | 3090.72±359.242a | 3182.87±110.541a |
| betT | K02168 | 329.67±77.643a | | 284.75±24.858a | 258.80±32.866a |
| desB | K04099 | 70.11±7.595a | | 65.74±10.574a | 89.94±12.334a |
| desA | K15064 | 124.22±9.595a | | 100.83±8.731a | 122.35±14.506a |
| gpx | K00432 | 721.33±64.643a | | 843.07±69.101ab | 615.99±9.904b |
| gumD | K13656 | 11.65±2.158a | | 13.63±6.319a | 8.13±1.851a |
| kup | K03549 | 1556.95±67.523a | | 1674.50±127.167a | 1642.63±63.306a |
| murA | K00790 | 3737.46±224.002a | | 3830.97±322.112a | 3954.28±92.110a |
| murC | K01924 | 2889.11±69.851a | | 2799.18±157.310a | 2745.94±63.712a |
| nhaA | K03313 | 1621.57±62.143a | | 1578.86±73.313a | 1586.03±21.474a |
| nhaB | K03314 | 47.87±20.294a | | 28.33±3.451a | 17.51±4.705a |
| otsB | K01087 | 1122.28±57.630a | | 983.02±83.421a | 1137.58±18.632a |
| nifQ | K15790 | 0.85±0.853a | | 5.37±3.445a | 1.32±1.322a |
| nifU | K04488 | 1962.21±47.286a | | 1806.47±134.166a | 1897.24±79.537a |
| proA | K00147 | 1861.49±93.391a | | 1859.93±100.786a | 1873.07±11.825a |
| proB | K00931 | 1509.64±49.742a | | 1630.53±98.695a | 1583.05±25.649a |
| proC | K00286 | 1695.76±54.621a | | 1676.51±84.654a | 1697.56±50.635a |
| prx | K24158 | 1261.86±52.479a | | 1084.65±39.029ab | 1209.81±31.294b |
| amoB | K10945 | 116.86±14.541a | | 134.90±26.532a | 148.56±9.774a |
| hsbA | K20978 | 12.35±7.991a | | 9.44±4.913a | 6.99±1.973a |
| rpoS | K03087 | 247.20±35.713a | | 240.89±37.476a | 281.39±15.181a |
| phzG | K20262 | 4.21±2.732a | | 15.47±12.453a | 6.54±5.341a |
| phzD | K20261 | 3.98±2.400a | | 16.85±8.180a | 0.00±0.000a |
| fepA | K19611 | 49.30±10.705a | | 125.52±94.863a | 29.43±6.134a |
| feoA | K04758 | 41.99±8.490a | | 107.17±83.278a | 33.46±10.586a |
| spoVFB | K06411 | 15.02±0.967a | | 15.31±4.826a | 14.52±4.438a |
| treS | K17311 | 21.63±1.789a | | 17.55±3.722a | 28.61±4.912a |
| trpB | K01696 | 3544.63±100.668a | | 3492.04±219.292a | 3494.62±120.813a |
| wza | K01991 | 668.90±87.373a | 709.15±178.416a | | 599.09±22.878a |
| etk | K16692 | 124.81±3.189a | 212.52±55.381a | | 118.25±12.899a |
| appA | K01093 | 3.91±1.810a | 3.64±2.033a | | 1.37±0.804a |
| ppsR | K09773 | 434.48±23.135a | 519.19±29.454a | | 469.35±37.543a |
| rpoP | K03059 | 20.69±8.758a | 31.25±9.403a | | 41.87±12.096a |

KO_ID: KEGG Orthology identifier

G1 (Soil samples from chemical fertiliser plot)

G2(Soil samples from biofertiliser plot)

G3(Soil samples from uncultivated bulk soil).

## Table S8: PERMANOVA results showing differences in microbial functional traits composition associated with the soil fertilisation systems.

|  | Df | Sum Of Sqs | R2 | F | Pr(>F) |
| --- | --- | --- | --- | --- | --- |
| Model | 2 | 0.0034 | 0.1005 | 0.5029 | 0.86 |
| Residual | 9 | 0.0307 | 0.8995 | - | - |
| Total | 11 | 0.0342 | 1 | - | - |

## Table S9: Alpha diversity indices of the functional genes across sample plots

| SAMPLEID | Shannon | Chao1 | Evenness |
| --- | --- | --- | --- |
| G1 | 2.83 ± 0.0065 | 32.75 ± 0.6292 | 0.812 ± 0.0053 |
| G2 | 2.85 ± 0.0332 | 34.00 ± 0.4082 | 0.810 ± 0.0094 |
| G3 | 2.82 ± 0.0063 | 32.25 ± 0.4787 | 0.813 ± 0.0044 |

G1 (Soil samples from chemical fertiliser plot)

G2(Soil samples from biofertiliser plot)

G3(Soil samples from uncultivated bulk soil).


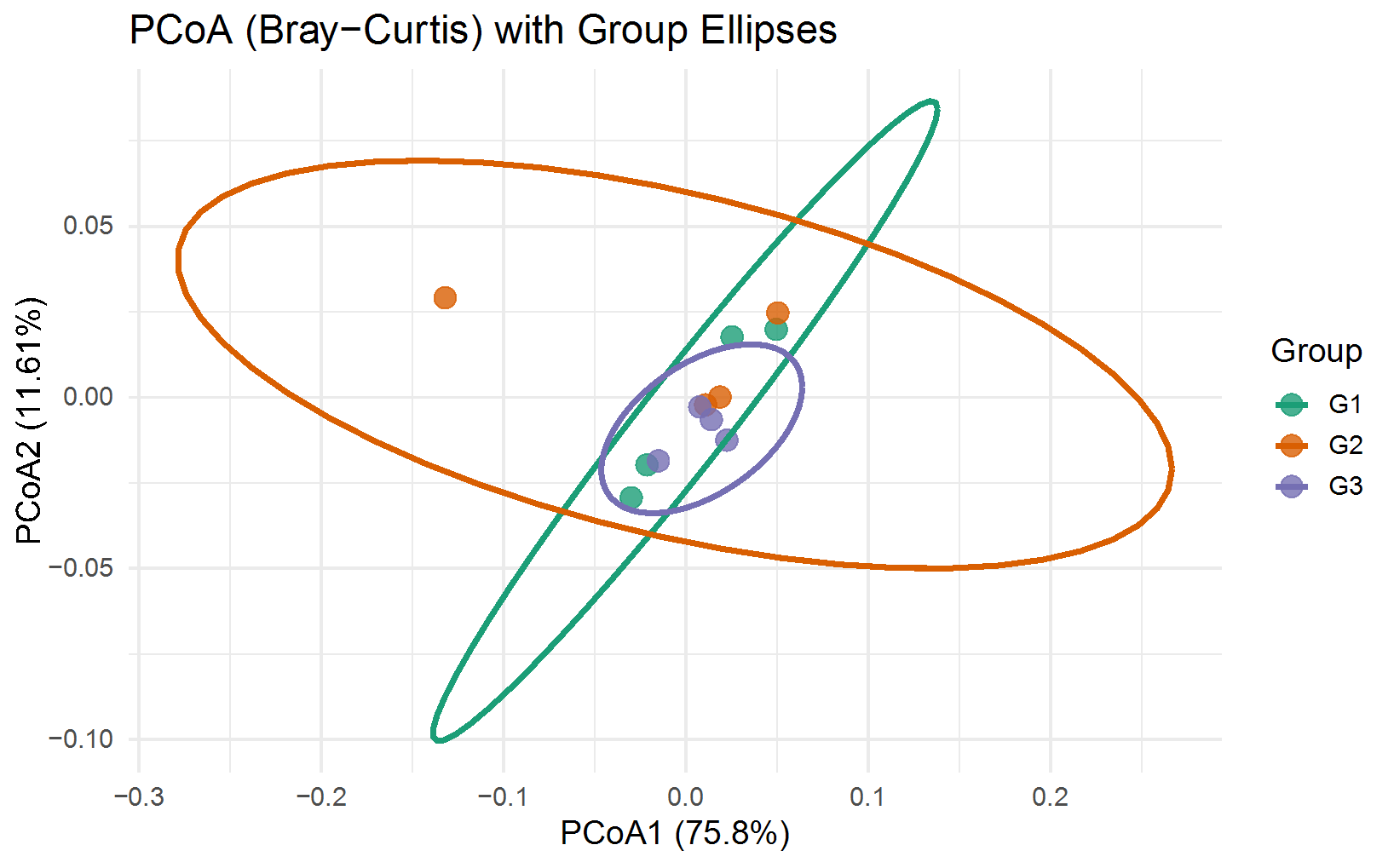


## Figure S1: PCoA graph of the functional genes’ abundance across sample plots

## Figure S2: Relative distribution of functional genes across the sample plots
